# Inhalation exposure to cigarette smoke and inflammatory agents induces epigenetic changes in the lung

**DOI:** 10.1038/s41598-020-67502-8

**Published:** 2020-07-09

**Authors:** Christopher L. Seiler, J ung Min Song, Delshanee Kotandeniya, Jianji Chen, Thomas J. Y. Kono, Qiyuan Han, Mathia Colwell, Benjamin Auch, Aaron L. Sarver, Pramod Upadhyaya, Yanan Ren, Christopher Faulk, Silvio De Flora, Sebastiano La Maestra, Yue Chen, Fekadu Kassie, Natalia Y. Tretyakova

**Affiliations:** 1 Department of Medicinal Chemistry, University of Minnesota, Minneapolis, MN 55455 USA; 2 Department of Veterinary Medicine, University of Minnesota, Minneapolis, MN 55455 USA; 3 Department of Biochemistry, Molecular Biology and Biophysics, University of Minnesota, Minneapolis, MN 55455 USA; 4 Minnesota Supercomputing Institute, University of Minnesota, Minneapolis, MN 55455 USA; 5 Department of Animal Science, University of Minnesota, Minneapolis, MN 55455 USA; 6 Genomics Center, University of Minnesota, Minneapolis, MN 55455 USA; 7 Institute for Health Informatics, University of Minnesota, Minneapolis, MN 55455 USA; 8 Biostatistics Core, University of Minnesota, Minneapolis, MN 55455 USA; 9 Masonic Cancer Center, University of Minnesota, 2231 6th Street SE, 2-147 CCRB, Minneapolis, 55455 USA; 10 Department of Health Sciences, University of Genoa, 16132 Genoa, Italy

**Keywords:** Lung cancer, Epigenetics

## Abstract

Smoking-related lung tumors are characterized by profound epigenetic changes including scrambled patterns of DNA methylation, deregulated histone acetylation, altered gene expression levels, distorted microRNA profiles, and a global loss of cytosine hydroxymethylation marks. Here, we employed an enhanced version of bisulfite sequencing (RRBS/oxRRBS) followed by next generation sequencing to separately map DNA epigenetic marks 5-methyl-dC and 5-hydroxymethyl-dC in genomic DNA isolated from lungs of A/J mice exposed whole-body to environmental cigarette smoke for 10 weeks. Exposure to cigarette smoke significantly affected the patterns of cytosine methylation and hydroxymethylation in the lungs. Differentially hydroxymethylated regions were associated with inflammatory response/disease, organismal injury, and respiratory diseases and were involved in regulation of cellular development, function, growth, and proliferation. To identify epigenetic changes in the lung associated with exposure to tobacco carcinogens and inflammation, A/J mice were intranasally treated with the tobacco carcinogen 4-(methylnitrosamino)-1-(3-pyridyl)-1-butanone (NNK), the inflammatory agent lipopolysaccharide (LPS), or both. NNK alone caused minimal epigenetic alterations, while exposure either to LPS or NNK/LPS in combination led to increased levels of global cytosine methylation and formylation, reduced cytosine hydroxymethylation, decreased histone acetylation, and altered expression levels of multiple genes. Our results suggest that inflammatory processes are responsible for epigenetic changes contributing to lung cancer development.

## Introduction

Lung cancer is responsible for 30% of all cancer deaths worldwide and is expected to kill 154,050 Americans this year, with over 80% of cases directly attributable to smoking^1^. Cigarette smoke contains over 60 known carcinogens, such as the tobacco specific nitrosamine 4-(methylnitrosamino)-1-(3-pyridyl)-1-butanone (NNK, 75 ng/cigarette)^2,3^, as well as non-genotoxic co-carcinogens including the inflammatory agent lipopolysaccharide (LPS, 120 ng/cigarette)^4^.

Chronic inflammation plays a central role in the pathogenesis of smoking-induced lung cancer^5^. Smoking is characterized by neutrophilic inflammation and reduced mucociliary clearance in the lung, which, at least in part, can be explained by exposure to LPS and other endotoxins^6^. When administered intranasally to laboratory mice, LPS induces an inflammatory response mimicking chronic obstructive pulmonary disease (COPD), a major risk factor for lung cancer development in smokers^7^. Furthermore, long-term exposure to LPS increases lung tumor size and multiplicity in A/J mice following treatment with NNK, supporting a role for inflammation in lung cancer etiology^7^. Anti-inflammatory agents such as aspirin and other NSAIDS have been suggested as potential chemopreventive agents for lung cancer^8^.

Smoking-induced lung tumors are characterized by genetic alterations in tumor suppressor genes and protooncogenes, as well as epigenetic changes which include deregulated DNA methylation, altered histone acetylation, and aberrant microRNA expression^9^. Aberrant DNA methylation patterns in malignant cells result in silencing of tumor suppressor genes, activation of protooncogenes, and decreased chromosomal stability^10^. These “epimutations” are thought to cooperate with genetic alterations to drive the malignant lung tumor phenotype^9^. However, the nature and the mechanistic origins of smoking-induced epigenetic deregulation remain largely unknown, limiting our understanding of cancer etiology and hindering the development of future treatments.

In the present work, we characterized epigenetic changes in the lungs of A/J mice exposed to cigarette smoke by inhalation for 10 weeks or intranasally treated with the tobacco carcinogen NNK and/or inflammatory agent LPS. Previous studies have shown that this treatment leads to pulmonary inflammation, atelectasis, emphysema, vascular alterations, bronchial hyperplasia, and alveolar bronchiolarization^11^. Our results revealed inflammation-driven changes in cytosine methylation and hydroxymethylation patterns indicative of an imbalance of DNA methylation/demethylation dynamics, which in turn give rise to a shift in histone acetylation marks and gene expression patterns that could contribute to initiation of lung cancer.

## Results

### Animal studies

**Figure 1:**
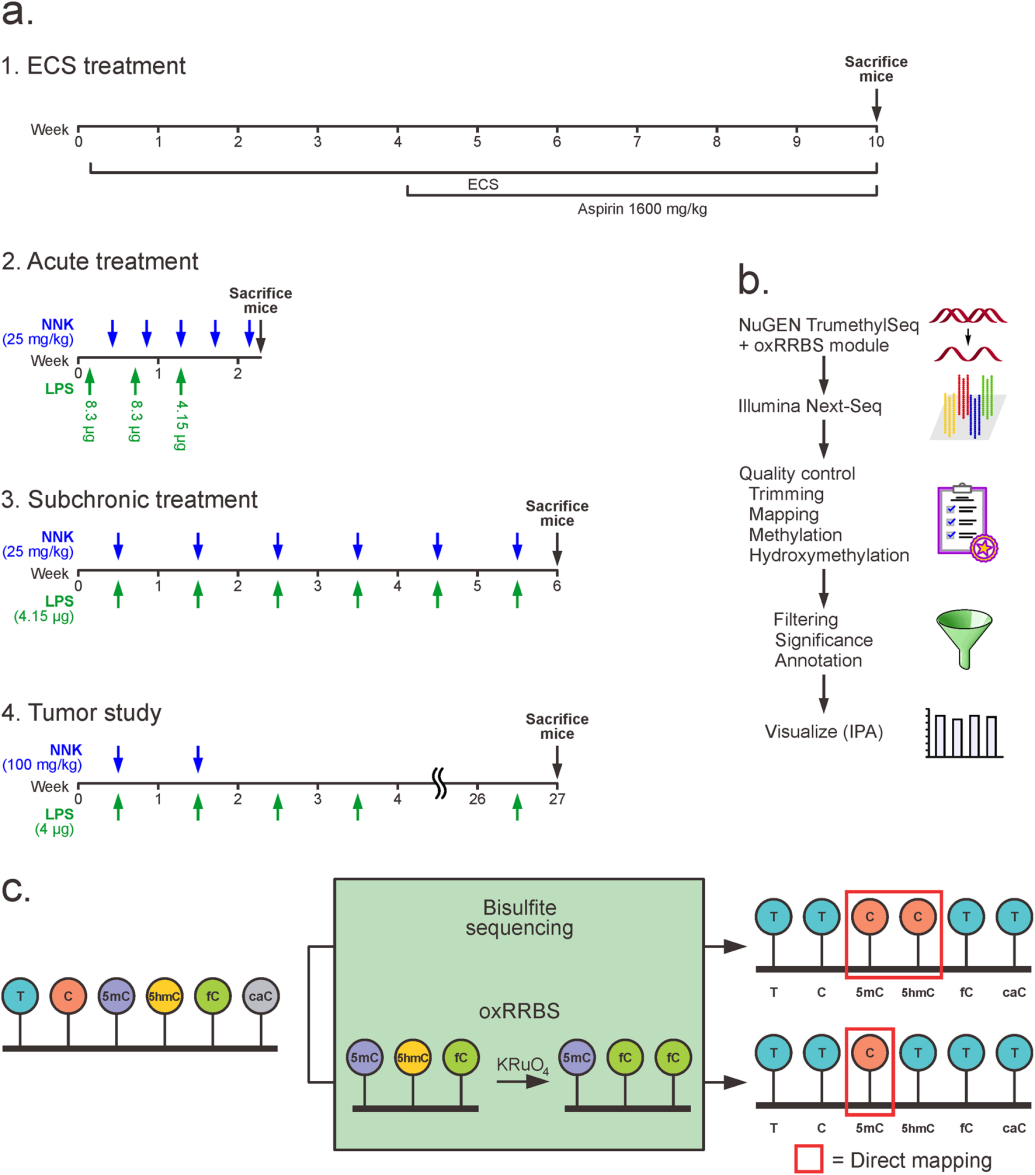
(**a)** Animal study design. 1, Exposure to environmental cigarette smoke (ECS) (N = 5). 2, Acute treatment with NNK and LPS (N = 3). 3, Subchronic treatment with NNK, LPS, and NNK/LPS (N = 3)^8^. 4, tumor study, (N = 3, tumors were pooled from 3 mice for each measurement).

Our experimental design included several mouse studies aimed to characterize epigenetic changes in the lung induced by exposure to cigarette smoke and its components (Fig. 1a), all conducted in A/J mice. In the smoking (ECS) study (panel **1** in Fig. 1a), A/J mice were treated with cigarette smoke for 10 weeks starting at birth with or without oral co-administration of the nonsteroidal anti-inflammatory agent acetylsalicylic acid (aspirin) on weeks 4–10^8^. In the acute treatment study (panel **2** in Fig. 1a), mice were exposed to the tobacco specific nitrosamine NNK (25 mg/kg, in 0.3 ml physiological saline solution, every three days) and/or the inflammatory agent lipopolysaccharide (LPS, intranasal instillation of 8.3 µg/mouse on days 1 and 5 and 4.15 µg/mouse on day 9) and either sacrificed on day 16 or allowed to recover for 1 week. In the sub-chronic study, animals were treated with NNK (25 mg/kg once a week) and/or LPS (4.15 µg/mouse) for 6 weeks as shown in panel **3** of Fig. 1a. Finally, **Study 4** (panel **4** in Fig. 1a) examined epigenetic changes in lung tumors. Mice were treated with two weekly doses of NNK (100 mg/kg) and exposed to LPS every week beginning with the first dose of NNK and until the termination of the study at week 27. Upon sacrifice of the mice, lungs were harvested, tumors on the surface of the lung counted, and some of them dissected for subsequent downstream assays. Histopathological analysis of the tumors harvested at week 27 showed that about 50% of the tumors had progressed to adenoma with dysplasia.

### Global epigenetic marks of DNA in lung DNA of A/J mice exposed to cigarette smoke

**Figure 2:**
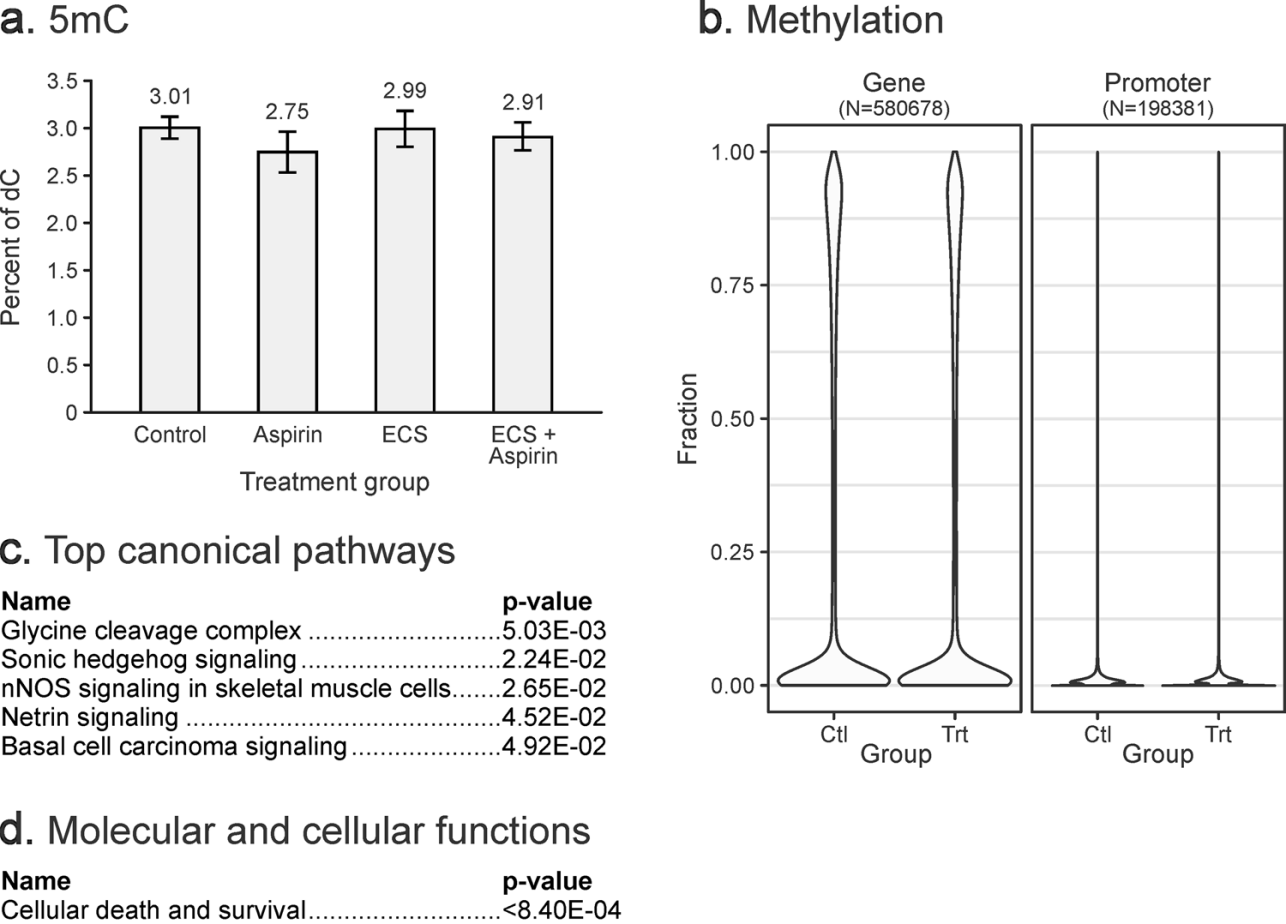
DNA methylation changes in lung DNA of female A/J mice (N = 5) exposed to ECS for 10 weeks with or without aspirin co-treatment. (**a**) Global cytosine methylation levels determined by isotope dilution HPLC–ESI–MS/MS. Values represent the mean ± standard deviation of 5 independent measurements. (**b**) Violin plots of methylation fractions of CpG sites in gene bodies and promoters, as assayed by RRBS/oxRRBS. Differential methylation data was input into Ingenuity Pathway Analysis (IPA). (**c**) Top canonical pathways associated with DMRs. (**d**) Molecular and cellular functions associated with DMRs.

**Figure 3:**
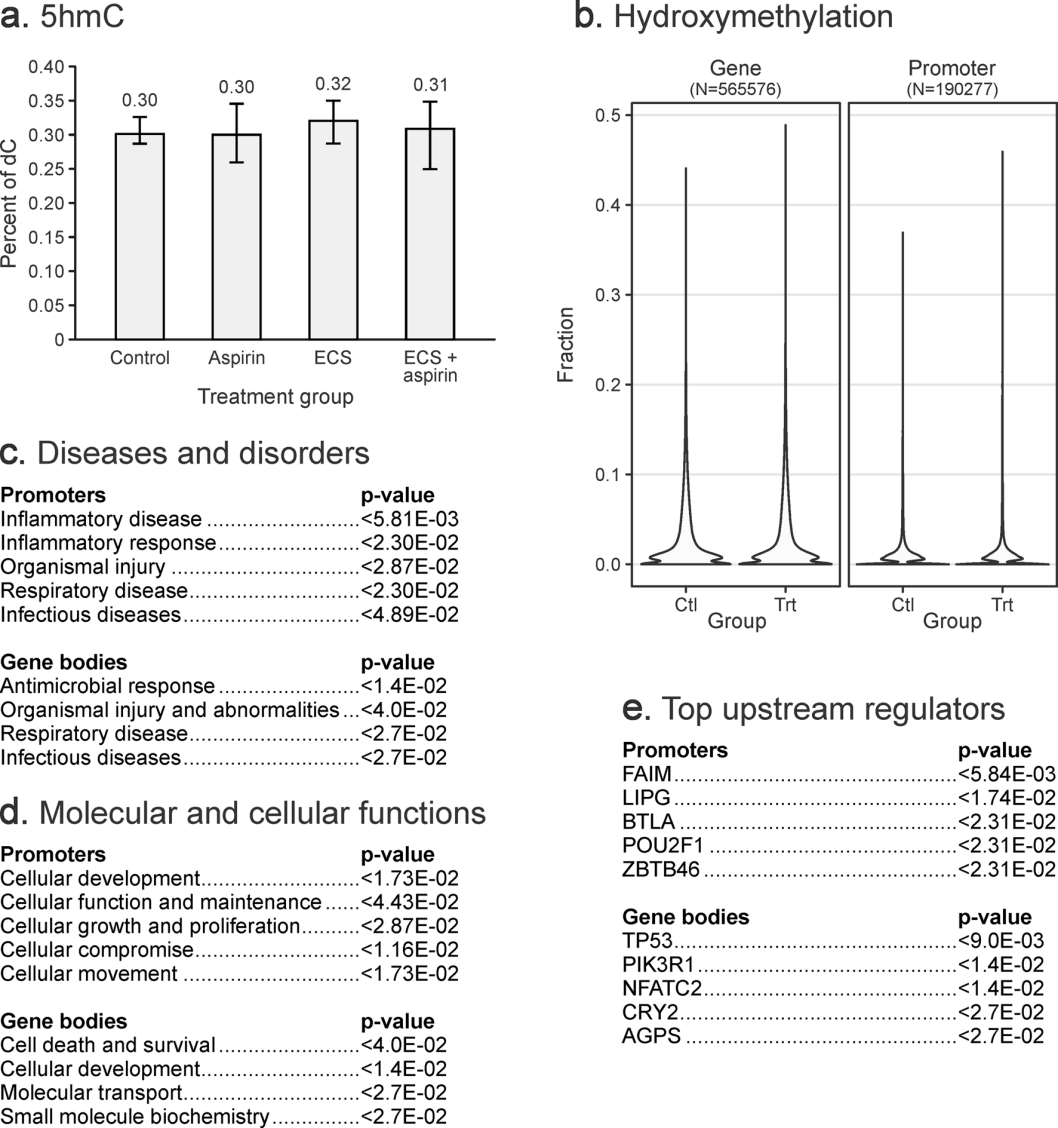
DNA hydroxymethylation changes in lung DNA of female A/J mice exposed to ECS for 10 weeks with or without aspirin co-treatment. (**a**) Global 5hmC levels as determined by isotope dilution HPLC–ESI–MS/MS. The values shown are mean ± standard deviation (N = 5). (**b)** Violin plots of hydroxymethylation fraction of all assayed CpG sites in gene bodies and promoters determined by RRBS/oxRRBS. Differential hydroxymethylation data was subjected to Ingenuity Pathway Analysis (IPA). (**c**) Top diseases and disorders associated with hydroxymethylation changes. (**d**) Top molecular and cellular functions associated with hydroxymethylation changes. (**e**) Top upstream regulators associated with hydroxymethylation changes.

A quantitative isotope dilution HPLC-ESI^+^-MS/MS methodology developed in our laboratory (Supplementary Methods, Figs. S1–S3) was used to quantify global levels of cytosine methylation, hydroxymethylation, formylation, and carboxylation in lung DNA of mice chronically exposed to cigarette smoke^12^. Total amounts of 5mC and 5hmC in genomic DNA isolated from lung tissues of A/J mice (2.8–3.0% of total cytosines being methylated, ~ 0.3% of total cytosines being hydroxymethylated, and 0.02–0.03% being formylated—see Figs. 2a, 3a, Supplementary Fig. S4) were comparable to previously published values for mouse lung^13^. HPLC-ESI^+^-MS/MS revealed that global 5mC, 5hmC, and fC concentrations in DNA isolated from lung DNA of A/J mice following whole-body exposure to environmental cigarette smoke (ECS) or ECS and aspirin for 10 weeks were similar to those of unexposed controls, suggesting that these treatments do not affect the overall cytosine methylation, hydroxymethylation, and formylation levels (see Figs. 2a, 3a, and Supplementary Fig. S4, respectively). The amounts of 5-carboxylcytosined in lung tissues were below our method’s limit of quantitation.

### Genome-wide changes in cytosine methylation and hydroxymethylation revealed by oxRRBS—NGS

Although the total amounts of 5mC, 5hmC, and fC in mouse lung DNA were unaffected by exposure to cigarette smoke (Figs. 2a, 3a, and Supplementary Fig. S4), this does not rule out potential localized changes at specific genomic loci. Therefore, reduced representation bisulfite sequencing (RRBS) and oxidative-RRBS (oxRRBS) were used to probe for site specific methylation and hydroxymethylation changes^14^. oxRRBS is a relatively new methodology that makes it possible to separately map 5mC and 5hmC in CpG rich regions of the genome (Fig. 1c)^14^. Standard RRBS employs bisulfite treatment to convert C to U, while 5mC resists deamination^15,16^. Unfortunately, this method does not distinguish between 5mC and 5hmC, therefore the majority of previously published studies actually report the sum of 5mC and 5hmC. In oxRRBS, separate mapping of the two epigenetic marks is achieved by selectively oxidizing 5hmC to fC using potassium perruthenate and separately performing bisulfite sequencing on oxidized and untreated DNA (Fig. 1c)^14^. Cytosine hydroxymethylation amounts at each genomic location were determined by subtracting the bisulfite sequencing signals obtained from oxidized sample (oxRRBS) from the signal generated upon standard bisulfite sequencing (RRBS) (Fig. 1c).

DNA isolated from lung tissues of female A/J mice exposed to ECS and control groups (4 animals per group) was prepared for sequencing using NuGEN Ovation RRBS Methyl-Seq with TrueMethyl oxBS modules^14^. Following sequencing, the data were screened for contaminants and low quality reads using FastQC, trimmed with TrimGalore, and mapped to the mouse mm10 genome with Bismark and Bowtie 2 (Fig. 1b). Cytosine methylation amounts were directly inferred from the oxRRBS signals using MethPipe^17^. To examine site-specific changes in levels of cytosine hydroxymethylation, we calculated the amounts of 5hmC present at each site by subtracting the signal in the oxRRBS data (5mC only) from the RRBS signals (5mC and 5hmC) using mlml (Fig. 1c)^18^.

**Figure 4:**
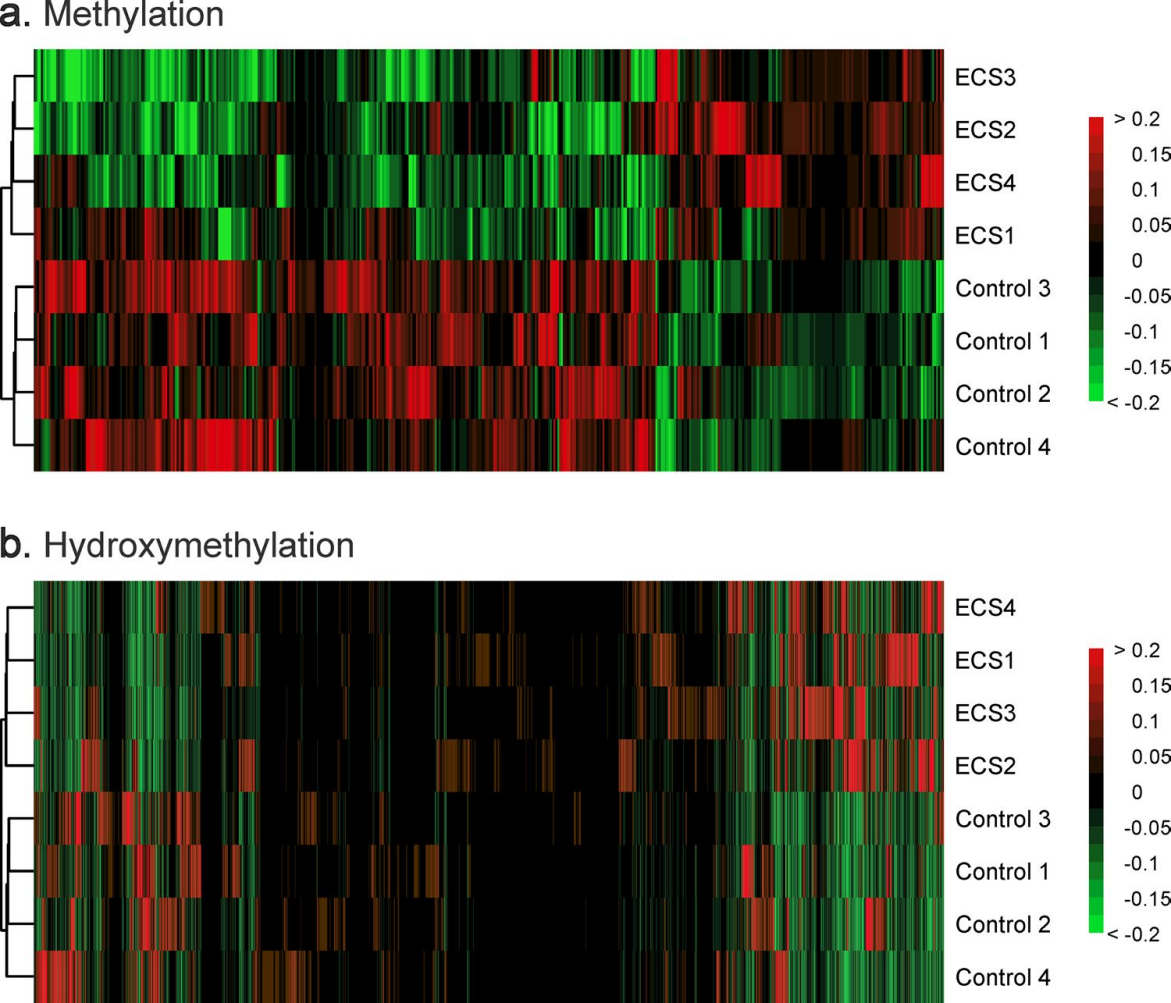
Heatmaps of CpG methylation and hydroxymethylation changes in DMRs and DhMRs of lung DNA of female A/J mice exposed to ECS for 10 weeks as revealed by RRBS and oxRRBS. Each row represents a separate sample (ECS 1–4, controls 1–4), while columns indicate individual CpG sites. Red color indicates levels above the mean value, while green color indicates the levels below the mean. The dendrogram shows the hierarchical clustering relationships among samples. Replicates of control and treated samples cluster together, revealing ECS-induced changes. (**a**) Mean centered methylation fractions of CpG sites in DMRs (483 CpGs). (**b**) Mean centered hydroxymethylation fractions in DhMRs (17,952 CpGs).

RRBS sequencing of DNA isolated from mouse lung tissue resulted in 20,490,683.5 (± 2,139,937.9) reads, while oxRRBS sequencing yielded 18,351,164.75 (± 2,402,886.9) reads. After filtering for non-CpG methylation/hydroxymethylation, sites in blacklisted regions (ENCODE file ENCFF547MET), and sites covered by fewer than 10 reads per sample, a total of 947,006 CpG sites were covered by both RRBS and oxo-RRBS data. We identified 58 differentially methylated regions (DMRs) and 1,902 differentially hydroxymethylated regions (DhMRs) (Fig. 4). DMR/DhMR was defined as a region that contained at least 3 modified CpG sites within 200 bp from each other with a false-discovery rate (Q-value) of less than 0.05. We have calculated the distance from each DMR/DhMR to its overlapped gene’s transcription start site (TSS) via Chipseeker^19^. If no gene was overlapped with a specific DMR/DhMR, the distance to its nearest TSS was reported (Supplementary Fig. S13). By plotting the distribution of distance from DMR/DhMR relative to transcription start site, we found that only around 15% DMRs were in the proximal TSS region (within 3 kb) while for DhMR this number was around 48% (Fig. S13).

Mean methylation differences at individual CpG sites between control and treatment groups ranged between 24.3% hypermethylation to 30.1% hypomethylation. Mean hydroxymethylation differences between control and treatment groups ranged from 31.5% increase to 29.5% decrease. As evident from the heat maps shown in Fig. 4, cytosine hydroxymethylation changes (Fig. 4b) took place at many more CpG sites across the genome as compared to methylation changes (Fig. 4a). However, the magnitude of the change was greater for CpG site methylation as compared to CpG hydroxymethylation (Fig. 4).

**Figure 5:**
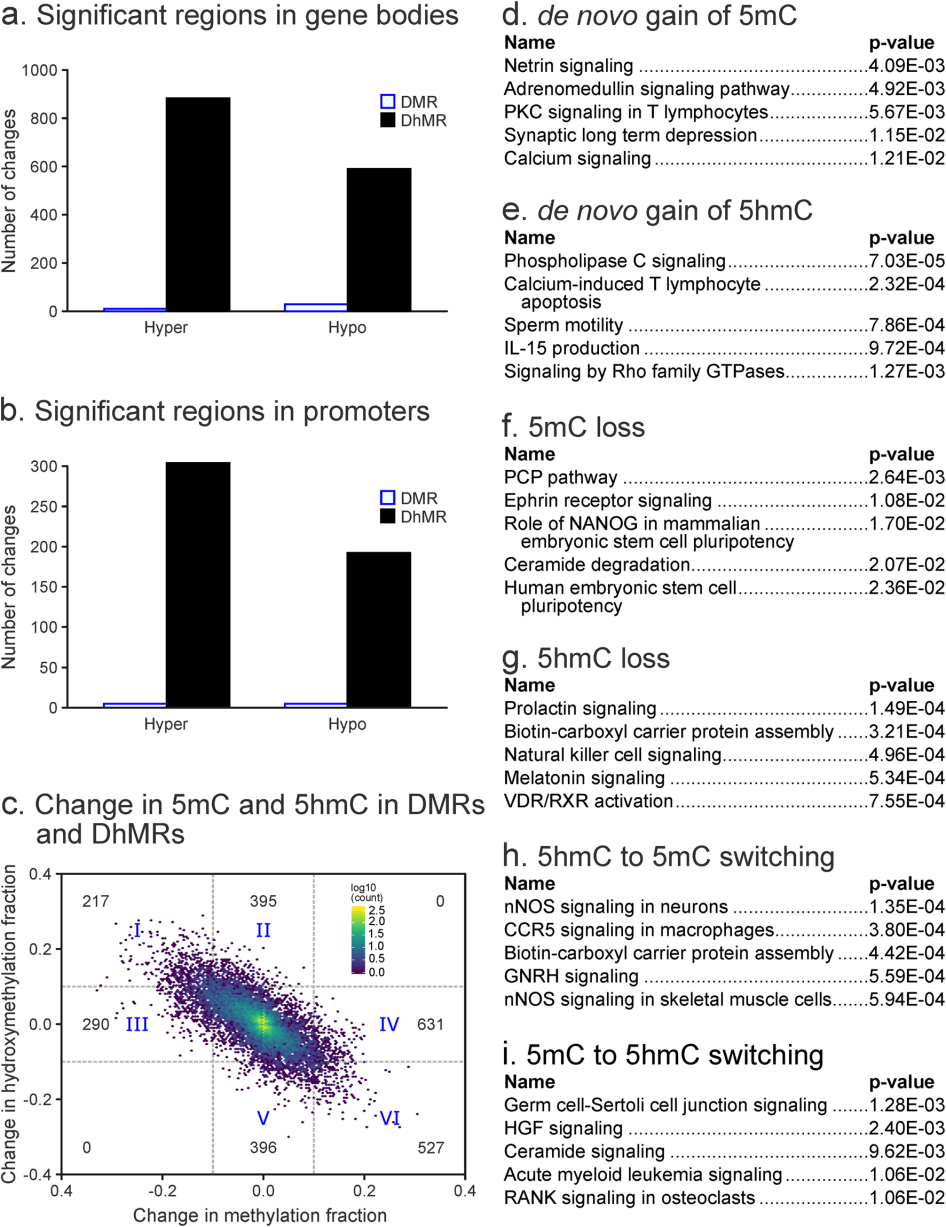
Site-specific methylation and hydroxymethylation changes in lung DNA of female A/J mice exposed to ECS for 10 weeks as revealed by RRBS and oxRRBS. Differentially hydroxymethylated regions in gene bodies (**a**) and promoters (**b**). Correlation of 5mC and 5hmC at CpG sites within DMRs and DhMRs, numbers represent the counts of sites (**c**). Top canonical pathways from IPA analysis of sites showing: de novo gain of methylation in DMRs (Δ5mC > 0.1 and − 0.1 < Δ5hmC < 0.1) (**d**), de novo gain of 5hmC in DHMRs (Δ5hmC > 0.1 and − 0.1 < Δ5mC < 0.1) (**e**), loss of 5mC (Δ5mC < − 0.1 and − 0.1 < Δ5hmC < 0.1) (**f**), loss of 5hmC (Δ5hmC < − 0.1 and − 0.1 < Δ5mC < 0.1) (**g**), switching from 5hmC to 5mC (Δ5hmC < − 0.1 and Δ5mC > 0.1) (**h**), switching from 5mC to 5hmC (Δ5hmC > 0.1 and Δ5mC < − 0.1) (**i**).

Initial data analyses revealed a characteristic bimodal enrichment of completely methylated CpG sites (methylation fraction close to 1) and completely unmethylated CpG sites (methylation fraction close to 0) within genomic regions (Fig. 2b, left panel). In contrast, the majority of CpG sites in promoter sequences were completely unmethylated (Fig. 2b, right panel). A total of 39 gene bodies and 9 promoters contained differentially methylated regions (Fig. 5a,b). Regions showing changes in cytosine methylation in promoter regions were enriched in the genes involved in glycine cleavage complex, nitric oxide synthase (NOS) signaling, and cell death and survival (Fig. 2c, d).

OxRRBS analyses revealed that the cytosine hydroxymethylation fraction was below 0.2 in both gene body and promoter regions (Fig. 3b). Cytosine hydroxymethylation changes were much more common than methylation changes, with 1,315 gene bodies, 108 promoters, and 4 enhancers containing differentially hydroxymethylated regions (Fig. 5a,b). Overall, the number of CpGs in differentially hydroxymethylated regions (DhMRs) greatly exceeded that of DMRs (17,952 vs 483 CpGs), although methylation changes were more pronounced (Figs. 4, 5a, b). Genes characterized by changes in cytosine hydroxymethylation upon exposure to ECS showed an enrichment for those participating in inflammatory disease, inflammatory response, and respiratory disease, while gene body hydroxymethylation was associated with antimicrobial response, organismal injury, and respiratory disease (Fig. 3c). Top molecular and cellular functions exhibiting aberrant hydroxymethylation included cellular development, cellular function, growth, movement, and proliferation (Fig. 3d), with tumor suppressor protein 53 (TP53), phosphoinositide-3-Kinase Regulatory Subunit 1 (PIK3R1), and nuclear factor of activated T Cells 2 (NFATC2) acting as top upstream regulators (Fig. 3e).

Decreased methylation in DMRs was strongly correlated with increased hydroxymethylation in DhMRs (Pearson correlation coefficient of − 0.63, *p* < 2.2e−16—see Fig. 5c), suggesting that 5mC was being converted to 5hmC. This inverse correlation remained regardless of whether all CpGs or only CpGs within DMRs/DhMR regions were considered. We were able to identify CpG sites that exhibited de novo gain of 5mC (quadrangle IV in Fig. 5c, d), de novo gain of 5hmC (quadrangle II in Fig. 5c, e), DNA methylation loss (quadrangle III in Fig. 5c, f), 5hmC loss (quadrangle V in Fig. 5c, g), sites with a shift from 5hmC to mC (quadrangle IV in Fig. 5c, h) and sites with a shift from mC to 5hmC (quadrangle I in Fig. 5c, i).

CpG sites characterized by de novo gain of 5mC (N = 631, quadrangle IV in Fig. 5c) included several members of the peroxisome-proliferator activated receptor (PPARG) pathway, which plays an important part in regulating cell proliferation, survival, and apoptosis^20^, as well as the engulfment and cell motility (ELMO1) signaling pathways, which are involved in phagocytosis and cell migration (See Supplementary Materials)^21^. The top canonical pathways included Netrin signaling, which is associated with immune response, influx of leukocytes, and inflammatory cytokine expression in the lung (Fig. 5d)^22^. Differentially modified CpG sites that underwent 5mC conversion to 5hmC (N = 217, quadrangle I in Fig. 5c) included T-cell factor/lymphoid enhancer-binding (Tcf7), nuclear factor NF-kappa-B p105 subunit (NFKB1) signaling^23^, and O-fucosylpeptide 3-beta-N-acetylglucosaminyltransferase (LFNG)^24^. CpG sites experiencing conversion of 5hmC to 5mC (N = 527, quadrangle VI in Fig. 5c) included MED1 (mediator of RNA polymerase II transcription subunit 1, nuclear receptor coactivator) signaling and were associated with cancer, inflammatory response, and organismal injury (Supplementary Materials)^25^. Overall, our results reveal a genome-wide deregulation of cytosine methylation and hydroxymethylation in the lungs of animals exposed to cigarette smoke, with genes involved in inflammation, transcriptional regulation, and cell growth preferentially affected by treatment.

### Epigenetic changes in lung tissues of A/J mice treated with the tobacco carcinogen NNK and the inflammatory agent LPS

To identify cigarette smoke components responsible for the observed epigenetic effects of ECS, A/J mice were intranasally treated with NNK, LPS, or both NNK and LPS in combination for 2 weeks (Study 2 in Fig. 1a). Isotope dilution capillary HPLC-ESI^+^-MS/MS (see above and Supplementary Figs. S1 and S2) was used to quantify global 5mC, 5hmC, fC, and caC in target (lung) and non-target tissues (kidney, brain), while pyrosequencing was utilized to detect methylation changes in specific genes, and qRT PCR was used to determine gene expression changes. We found that the global genomic levels of 5mC and 5hmC in mouse lung were essentially unchanged following two-week treatment with NNK (Supplementary Fig. S5). In contrast, global 5hmC concentrations were significantly lower in lung tissues of mice treated with LPS (*p* < 0.05) and the group treated with LPS and NNK in combination (0.13 ± 0.012% vs 0.071 ± 0.008%, *p* = 0.025) (see Supplementary Fig. S5). Global 5hmC levels returned to the original values 7 days post-treatment (Supplementary Fig. S5). In contrast, no significant changes in global DNA marks were seen in mouse brain and kidney DNA (Supplementary Fig. S6). These results provided initial evidence that NNK alone had little effect on epigenetic marks of DNA, while LPS treatment reduced the global levels of cytosine hydroxymethylation (5hmC) in the target tissue (lung), but not in non-target (brain, kidney) tissues.

**Figure 6:**
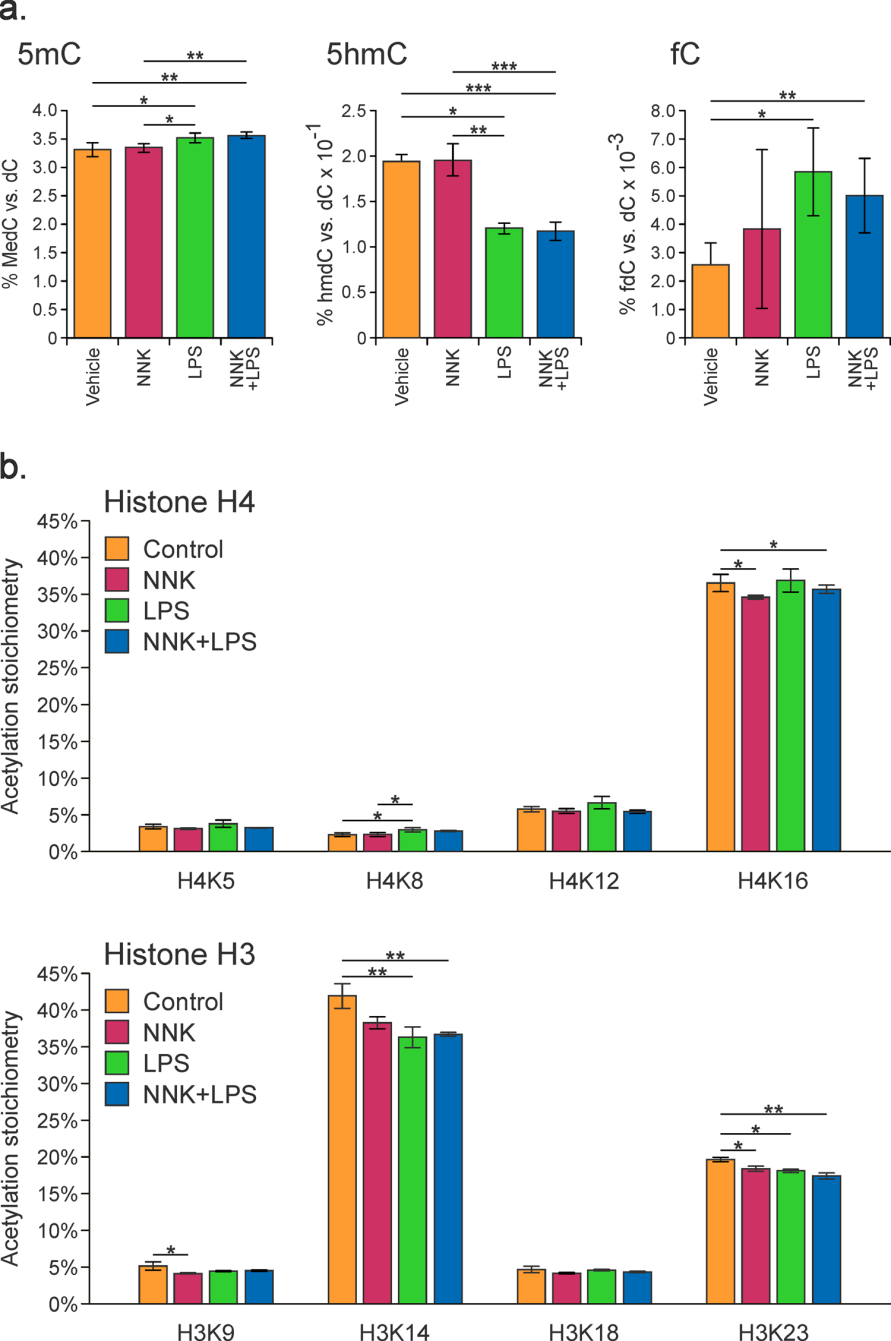
Global epigenetic changes observed in the lungs of female A/J mice treated with NNK, LPS, or both in combination for a length of 6 weeks (Study 3 in Fig. 1a). (**a**) Global levels of 5mC, 5hmC, and fC. (**b**) Site-specific histone lysine acetylation stoichiometry changes. Data represent an average value ± SD of at least 3 animals. Statistical significance was evaluated between treated and control samples using a two-sided Student’s t-test (*p < 0.05, **p < 0.01).

More pronounced epigenetic changes in the lung were observed when A/J mice were treated with NNK, LPS or NNK + LPS for a longer period (6 weeks) (subchronic treatment, Study 3 in Fig. 1a). HPLC-ESI^+^-MS/MS analyses revealed a small but significant increase in global amounts of 5mC in lung DNA of mice treated with LPS alone (3.32 ± 0.12% vs 3.52 ± 0.09%, p < 0.05) or NNK/LPS in combination (3.32 ± 0.12% vs 3.56 ± 0.05%, *p* < 0.05) (Fig. 6a). Furthermore, global 5hmC levels were significantly decreased in lung DNA of animals treated with NNK/LPS or LPS only (0.13 ± 0.009% C and 0.12 ± 0.006%, respectively, as compared to 0.19 ± 0.007% in control animals, *p* < 0.01) (Fig. 6a). An opposite trend was observed for global fC, which was elevated in LPS and NNK/LPS treatment groups 0.0058 ± 0.0015% (*p* = 0.05) and 0.0058 ± 0.0003% C (*p* < 0.01), respectively, as compared to 0.0026 ± 0.0007% in control (Fig. 6a). These changes were lung specific (Supplementary Fig. S7). Non significant alterations in global 5mC or 5hmC levels were observed in lung DNA of animals treated with NNK alone (*p* = 0.66 and 0.48, respectively, Fig. 6a).

Pyrosequencing revealed site specific changes in methylation of *CDH13* (2–23%), *Ahrr* (5–24%), *DAPK1* (5–23%), *Tet1* (5–46%), and *Rassf1* (9–14%) genes in animals treated with LPS (Supplementary Fig. S8). These five genes were selected based upon their respective relevance in lung cancer^26–28^. Aryl hydrocarbon receptor repressor (*Ahrr*) acts as a tumor suppressor gene in several types of cancer cells; *Ah*rr silencing is associated with exposure to cigarette smoke and lung cancer development^27^. The methylation level of the *Ahrr* gene (intron 1) was measured at 9 CpG sites, revealing small increases following treatment with NNK or LPS treatment alone (0.3–2.5%). Similarly, increases in *Ahrr* methylation were observed at CpG 3 (3%, *p* < 0.05), CpG 4 (2.8%), CpG 6 (2.1%), and CpG 9 (3.3%) of mice subjected to combined treatment with NNK and LPS.

Death-associated protein kinase (*DAPK1*) promoter methylation has been shown to correlate with clinicopathological and prognostic features in non-small cell lung cancer patients^29,30^. Pyrosequencing analysis of five CpG sites within *DAPK1* gene (exon 2) revealed an increase in methylation at all five CpG sites in animals co-treated with NNK and LPS (Supplementary Fig. S8). Upon co-treatment with NNK and LPS, methylation levels of *DAPK1* exon 2 increased by 0.8%, 1.6%, 1.5%, and 5.3% (*p* < 0.01) for CpG sites 1, 2, 3, and 4, respectively.

Cadherin 13 (*CDH13)* is a calcium dependent adhesion molecule important for cell–cell adhesion, and aberrant *CDH13* methylation is associated with non-small cell lung cancer^26,31,32^. Methylation levels of CpGs 1, 3, and 4 within *CDH13* intron 1 were increased by 3.2% (*p* < 0.05), 1.5%, and 4.8% (*p* < 0.01) following treatment with LPS alone and by 5.2% (*p* < 0.01), 2.5%, and 5.5% (*p* < 0.01), respectively, in animals treated with both NNK and LPS (Supplementary Fig. S8).

*RASSF1* gene is a potential tumor suppressor required for death receptor-dependent apoptosis^33^. Following treatment with NNK, LPS, or both, methylation levels of CpG 3 in Rassf1 in the lung were increased by 3.9% (*p* < 0.05), 3.7% (*p* < 0.05), and 4.5% (*p* = 0.01), respectively, while CpG 4 methylation increased 0.9% and 0.5% in NNK and NNK/LPS groups (Supplementary Fig. S8).

*Tet1* codes for ten eleven translocation dioxygenase 1, which is involved in active DNA demethylation by converting 5mC to 5hmC and further to formyl-C and carboxy-C^34^. Pyrosequencing detected very small methylation changes in the *Tet1* promoter, with NNK treatment causing a 0.3%, 0%, 0.1%, and 1.3% increase at CpGs 1, 2, 3, 4 and 1.2% decrease at CpG 5, which did not reach statistical significance (Supplementary Fig. S8). LPS treatment caused an increase of *Tet1* promoter methylation by 0.6%, 0.2%, 0.5% at CpGs 1, 2, 4, while CpG 3 and 5 exhibited 0.1% and 4.2% decrease, respectively (*p* = 0.01, Supplementary Fig. S8). Combined NNK + LPS treatment caused increased methylation of *Tet1* promoter (1.6, 1.2, 1.0, 1.5, 1.1, and 3.5% at GpGs 1, 2, 3, and 4 (*p* < 0.05), except for CpG 5, which showed a 1.6% decrease (Supplementary Fig. S8).

Overall, pyrosequencing revealed small, but statistically significant increases in methylation of *Ahrr, Dapk1, Cdh13*, and *Tet1*, but not *Rassf1,* in lung DNA of A/J mice treated with LPS or LPS/NNK in combination. Animals treated with NNK showed smaller methylation changes (Supplementary Fig. S8), revealing a key role of inflammation in inducing epigenetic changes in the lung.

### Global histone acetylation

We next examined whether cigarette smoke components affected histone marks in lung tissues of animals treated with NNK, LPS, or both. Specifically, we used a mass spectrometry based quantitative methodology developed in our group^35^ to examine acetylation stoichiometry of H3K14, H3K23, and H4K16 in control and treated mice. These transcriptional activation marks are strongly associated with active gene expression^36^. As shown in Fig. 6b, co-treatment with NNK and LPS led to the overall down-regulation of lysine acetylation within histones H3 and H4 in the lung. More specifically, acetylation on histones H3K14 and H3K23 was significantly decreased upon treatment with NNK, LPS alone, or NNK/LPS, while H3K18 acetylation was unchanged (Fig. 6b). Histone H4 acetylation was also downregulated by NNK and LPS treatment, but to a lesser extent than H3. NNK treatment alone and the combination of NNK/LPS treatment significantly reduced H4K16 acetylation, but did not affect other histone H4 N-terminal acetylation (Fig. 6b).

### Gene expression levels in lung tissues of NNK/LPS treated mice.

To determine whether exposures to LPS and NNK led to transcriptome changes in the lung, we examined the expression levels of *Tet1*, *Tet2*, *Tet3*, *Dapk1*, *Gata2*, *Cdh13*, *Prdm2*, *Rassf1*, and *Runx3* in the lung tissues of A/J mice treated with NNK, LPS, or both for a total of 9 weeks (Supplementary Fig. S9). These genes were selected based on their role in DNA demethylation (*Tet1, Tet2, Tet3*) and in lung cancer etiology (*Dapk1*, *Gata2*, *Cdh13*, *Prdm2*, *Rarβ*, *Rassf1*, and *Runx3)*^37–39^*.* Further, pyrosequencing revealed methylation changes in *Dapk1, Cdh13*, *Tet1*, and *Rassf1* following similar treatment (Supplementary Fig. S8). We found that *Tet1* gene expression was decreased in lung tissues of mice treated with NNK, LPS, or both, while the expression levels of *Tet* isoforms 2 and 3 were unaffected (Supplementary Fig. S9). NNK/LPS treated animals showed more pronounced changes in *Tet1* expression levels as compared to the NNK only group (*p* < 0.05, see Supplementary Fig. S9). These results are consistent with increased methylation of *Tet1* promoter (Supplementary Fig. S8) and decreased global levels of 5hmC in genomic DNA of exposed animals (Fig. 6a). In addition, significant changes in gene expression levels of tumor suppressor genes *Cdh13*, *Dapk1*, *Gata2*, *Prdm2*, and *Rassf1* were observed (Supplementary Fig. S9). Expression levels of *Cdh13* decreased threefold in both groups treated with LPS, but not in the NNK only group (Supplementary Fig. S9). Similarly, the expression levels of death-associate protein kinase 1 (*Dapk1*) showed a twofold decrease in both LPS treated groups, but was unchanged in the NNK only group (Supplementary Fig. S9). For the zinc-finger transcription factor *Gata2*, mRNA levels were reduced in each treatment group (Supplementary Fig. S9), with the most significant drop in the NNK/LPS co-treatment group (*p* < 0.05). The mRNA levels for PR Domain containing protein 2 (*Prdm2*) decreased slightly with treatment relative to control (Supplementary Fig. S9). Interestingly, expression levels of the retinoic acid receptor beta (*Rar-β*) were elevated upon treatment with NNK, but dropped in LPS alone and NNK/LPS groups (Supplementary Fig. S9). A small decrease in the expression of the Ras association domain family member 1 (*Rassf1*) was observed in all treatment groups (p < 0.05) (Supplementary Fig. S9). In contrast, expression levels of Runt related transcription factor 3 (*Runx3*) were elevated in both LPS treated groups, but unchanged in the NNK only group. Overall, significant changes in expression levels of many cancer associated genes were observed in the lung of mice treated with LPS to induce inflammation, while the effects of NNK alone on gene expression were relatively small.

### Epigenetic changes in NNK/LPS induced lung tumors

**Figure 7:**
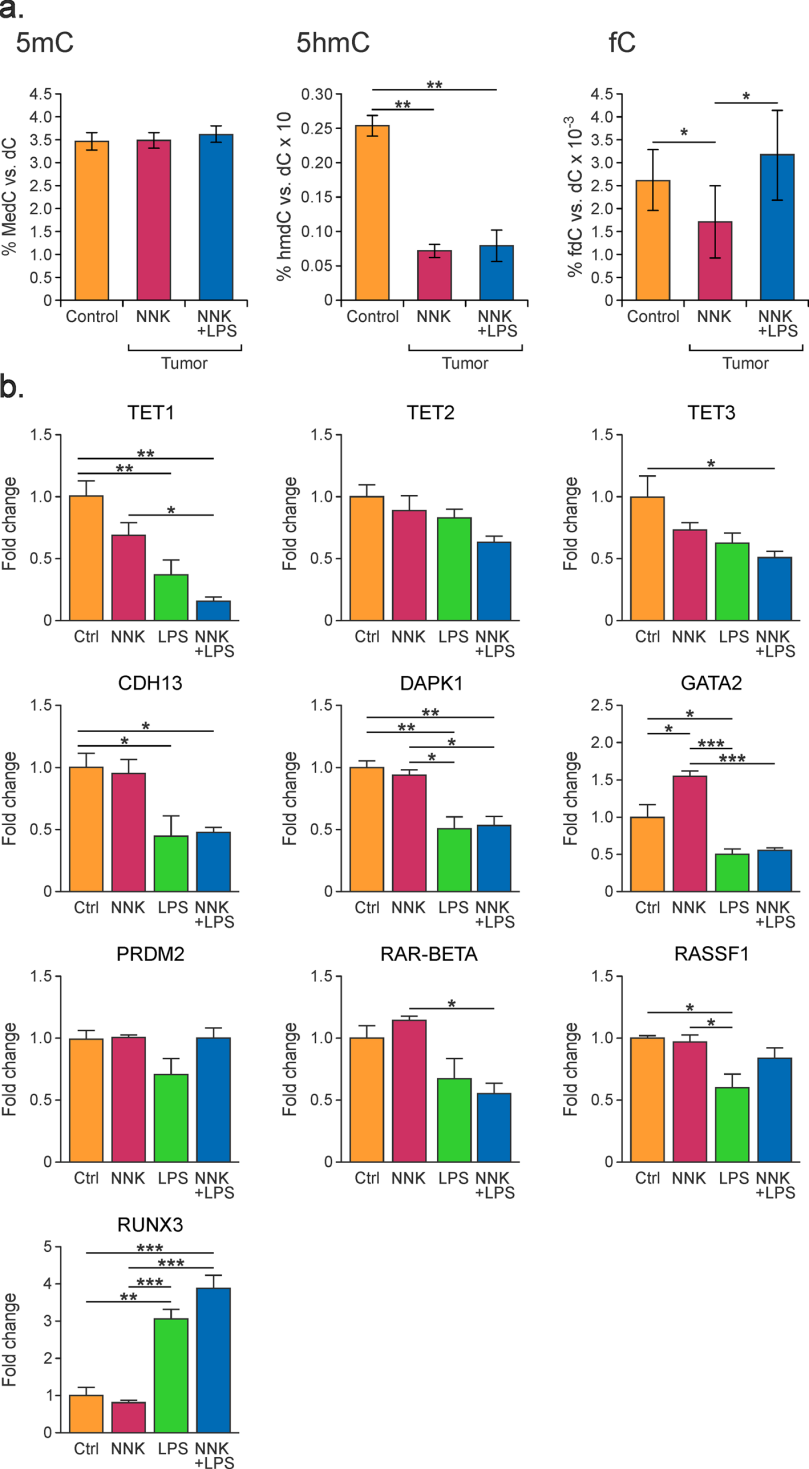
Global levels of 5mC, 5hmC, and fC in DNA isolated from lung tumors of female A/J mice (6 weeks of age) treated intraperitoneally with NNK, intranasally with LPS, or in combination. (**a**) Global epigenetic levels of 5mC, 5hmC, and fC. Data are expressed as percent of dC and represents mean values ± SD of at least three animals. (**b**) Gene levels of TET proteins and tumor suppressor genes changes in lung tumors of A/J mice. Data were calculated from qRT-PCR using the ΔΔCt method ± SD with three biological and three technical replicates.

To determine whether early tobacco carcinogen-induced epigenetic changes persist throughout lung cancer development, 5mC, hmC, and fC were quantified in lung tumors induced by NNK/LPS treatment (**study 4** in Fig. 1a). Tumors (≥ 100) isolated from the lungs of five A/J mice that had been treated with either NNK alone or NNK/LPS for 27 weeks were excised and pooled^7^. 5mC, 5hmC, and fC were quantified by isotope dilution HPLC-ESI^+^-MS/MS as described above. The values were compared to non-tumor DNA from lung tissues of control mice of the same age. We found that while the global levels of 5mC remained relatively stable across treatment groups (3.5 ± 0.2–3.6 ± 0.2% of total Cs, *p* = 0.15), both 5hmC and fC levels were altered in tumors (Fig. 7a). Cytosine hydroxymethylation decreased threefold in DNA extracted from NNK- and NNK/LPS-induced tumors (0.07 and 0.08%, respectively), as compared to 0.25 ± 0.015% of total Cs in normal lung tissue (*p* < 0.0001 and 0.0001, respectively, see Fig. 7a). Global amounts of fC showed a small decrease in NNK-induced tumors (0.0026 ± 0.0007 vs 0.0017 ± 0.0008% of total Cs, *p* = 0.027) and were increased in tumors induced by NNK/LPS treatment (0.0026 vs 0.0032 ± 0.001%), although this difference was not statistically significant (Fig. 7a). Overall, global amounts of 5mC, 5hmC, and fC in lung tumors exhibited the same overall trend as early changes observed in lung tissues of LPS/NNK treated animals prior to tumor formation (compare to Fig. 6a).

Methylation specific PCR analyses were conducted to determine whether global methylation and hydroxymethylation changes in tumors are accompanied by altered promoter methylation and hydroxymethylation of tumor suppressor genes. We found that the *Dapk1* promoter was unmethylated in all samples, while the methylation of *Cdh13*, *Runx3*, and *Gata2* promoters was unchanged between tumors and controls. However, *Rar-β* promoter was methylated only in the treated group but not in control lung (Supplementary Fig. S10). These results indicate that with the exception of *Rar-β,* promoter methylation status did not correlate with gene expression.

To understand whether initial inflammation-induced changes in gene expression in the lung persist upon tumor formation, mRNA expression analyses were repeated in lung tumors of A/J mice formed 22 or 44 weeks post treatment with NNK, LPS, or with NNK/LPS. Reduced *Tet1*, *Tet3, Cdh13*, *Dapk1*, *Gata2, Rarβ, and RASSF1* gene expression was observed in tumors, while the expression of *Runx3* was increased (22-week tumors—Fig. 7b, and 44-week tumors—Supplementary Fig. S11). These results support the notion that inflammation mediated changes in gene expression contribute to carcinogenesis in the A/J mouse model of lung cancer.

## Discussion

Chronic inflammation is a well-known risk factor for tumor development as reflected in a strong association between inflammatory bowel disease and colon cancer, *H. Pylori* induced inflammation and gastric cancer, and chronic obstructive pulmonary disease (COPD) and lung cancer^40,41^. COPD diagnosis doubles the risk of lung cancer in smokers, and long term use of anti-inflammatory agents reduces cancer risk^42^. While the exact mechanisms by which inflammation contributes to lung tumor development are unknown, early epigenetic changes associated with inflammation have been proposed to play a key role in this process^5^.

The main goal of the present study was to characterize epigenetic changes in the lung and in lung tumors following exposure to cigarette smoke and its components. A well-established mouse model of smoking induced lung cancer (A/J mouse) was employed^7^. We have previously reported that whole body exposure of A/J mice to ECS for 10 weeks led to pulmonary inflammation, atelectasis, emphysema, vascular alterations, bronchial hyperplasia, alveolar bronchiolarization^11^, and caused a downregulation of pulmonary microRNAs^43^. In the present work, A/J mice were exposed to ECS or its components (inflammatory agent LPS and tobacco carcinogen NNK) in order to identify the mechanisms and the dynamics of smoking-induced epigenetic deregulation (Fig. 1a)^8^. LPS dose (4.5 μg/mouse) corresponds to the LPS amounts delivered to the human lung by smoking of ∼ 25 cigarettes^6,44^. To reveal global and loci-specific changes in cytosine methylation and hydroxymethylation in the lung due to LPS treatment, genomic DNA was subjected to mass spectrometry analyses and bisulfite sequencing via next generation sequencing (NGS)-based methods, RRBS and oxRRBS. We further elucidated the epigenetic changes in animals treated with cigarette smoke components using pyrosequencing and qRT-PCR of tumor suppressor genes. Finally, the analyses were repeated for NNK/LPS induced lung tumors.

Exposure of laboratory mice to environmental cigarette smoke for 10 weeks induced significant epigenetic changes in the lung. These changes affected both gene bodies and promoter regions of genes. We identified 58 differentially methylated regions, which were enriched in genes participating in glycine cleavage complex, NOS signaling, and cell death and survival. RRBS/oxRRBS revealed 1,902 differentially hydroxymethylated regions, mostly in gene bodies but also in promoters. On average, hydroxymethylation changes were of a smaller magnitude as compared to changes in cytosine methylation. ECS-induced changes in cytosine hydroxymethylation showed an enrichment for inflammation pathways, respiratory disease, infectious diseases, and organismal injury response pathways. Overall, differentially hydroxymethylated regions were smaller in size than regions with altered methylation and were more widely distributed across the genome.

Decreased methylation in DMRs was correlated with increased hydroxymethylation in DhMRs, consistent with the interrelationship between the two epigenetic marks. Of those, 217 CpG sites experienced a shift from 5hmC to 5mC, 290 experienced de novo cytosine methylation, and 527 CpG sites with 5mC being converted to 5hmC (Fig. 5c). Overall, our results indicate that exposure to cigarette smoke induces site-specific changes in cytosine methylation and hydroxymethylation, and these changes take place preferentially within genes involved in inflammatory processes, cell migration, and cell proliferation.

To identify epigenetic changes in the lung associated with exposure to tobacco carcinogen NNK and inflammation, A/J mice were intranasally exposed to NNK, the inflammatory agent LPS, or both for 2 or 6 weeks (Fig. 1a). LPS is known to induce inflammation and emphysematous changes in the lung and NNK enhances this effect, while NNK treatment alone does not induce emphysema^44,45^. Isotope dilution HPLC-ESI^+^-MS/MS revealed a significant increase in global cytosine methylation accompanied by a decrease in cytosine hydroxymethylation in animals exposed to LPS or a combination of NNK and LPS, but not in mice treated with NNK alone (Fig. 6a). In mice treated with LPS for 2 weeks, a 33% decrease in global 5hmC levels was observed (Supplementary Fig. S5b), while changes in 5mC and fC required longer exposure (6 weeks, Supplementary Fig. S5a and Fig. 6a). Therefore, global DNA hydroxymethylation changes may serve as an early sensor of epigenetic deregulation.

Smoking-mediated early epigenetic changes in the lung observed in our study are likely to be triggered by inflammation. Indeed, lung tissues of mice treated with the tobacco carcinogen NNK alone exhibited minimal changes in DNA and histone marks, while exposure to the inflammatory agent LPS exhibited decreased global cytosine hydroxymethylation, increased methylation of tumor suppressor genes *DAPK1*, *CDH13*, *Rassf1*, *Tet1,* and *AHRR* (Supplementary Fig. S8), and decreased the levels of expression of these genes as revealed by qRT-PCR analyses (Supplementary Fig. S8)^26,27,31,32,46–51^. These results are significant because hypermethylation-induced decreases of expression of *Rassf1*, *CDH13* and *DAPK1* have been previously linked to lung cancer risk^26,33,52^.

To establish whether the early epigenetic changes induced by NNK/LPS persist during lung tumor development, DNA and RNA isolated from lung tumors of A/J mice were subjected to HPLC-ESI^+^-MS/MS and qRT-PCR analyses. We found that NNK/LPS induced lung tumors from 22 week long treatment were characterized by a large global decrease in 5hmC and decreased levels of expression of *Tet1*, *Tet3, Cdh13*, *Dapk1*, *Gata, Rarβ,* and *RASSF1* (Fig. 7). A similar trend was observed in lung tumors collected after 44 weeks of treatment with NNK/LPS (Supplementary Fig. S11).

To our knowledge, our study is the first to map smoking-induced changes in cytosine methylation and hydroxymethylation across the genome. While many previous reports characterized the alterations in cytosine methylation patterns in lung DNA of smokers and smoking-induced lung tumors^48^, traditional bisulfite sequencing does not distinguish between 5mC and 5hmC^53,54^. As 5hmC is at least 100-fold less abundant in the genome than 5mC, standard bisulfite sequencing methods are likely to overlook any hydroxymethylation changes.

Our results demonstrate that inflammation in the lung due to smoking and/or chronic obstructive pulmonary disease (COPD) alters the global epigenetic landscape of cytosine methylation, cytosine hydroxymethylation, and histone acetylation, potentially predisposing pulmonary cells to the onset of tumorigenesis. The epigenetic changes in the lung precede the formation of tumors, but appear to persist through tumor development and potentially contribute to lung cancer etiology. Our ongoing work focuses on characterizing epigenetic changes in specific cell types^12^, functional studies to define the contributions of specific epigenetic events to cancer etiology, and the development of epigenetic modulators that could be used in lung cancer chemoprevention and treatment.

## Methods

### Animal studies

All animal studies were conducted in female A/J mice. In the smoking study, newborn animals (4 per group) were exposed to environmental cigarette smoke (ESC) for 10 weeks, while the control group was treated with filtered air. In the acute and subchronic NNK/LPS exposure studies, mice (6 weeks of age, 3 per group) were treated intraperitoneally (IP) with NNK (25 mg/kg) and/or intranasally with LPS (4.15 or 8.3 µg). For the lung tumor study, mice were treated IP with NNK (100 mg/kg, in 0.3 ml PBS) once a week for two weeks and/or intranasally with LPS (4 µg) once a week until week 27.

Female A/J mice were obtained from the Jackson Laboratory (Bar Harbor, ME) and housed in specific-pathogen-free animal quarters at Research Animal Resources, University of Minnesota Academic Health Center. All animal experiments were performed according to the U.S. National Institutes of Health (NIH) Guide for the Care and Use of Laboratory Animals and was approved by the Institutional Animal Care and Use Committee, University of Minnesota. Details of the animal studies are given in Supplementary Methods and are illustrated in Fig. 1a.

### HPLC-ESI-MS/MS quantitation of global of 5mC, 5hmC, fC, and caC

Genomic DNA was extracted from lung, kidney, brain tissues and lung tumors using an IBI-Mini Genomic DNA Kit. DNA (2–10 µg) was spiked with ^13^C_10_^15^N_2_-5-methyl-2′-deoxycytidine (1 pmol), 5-hydroxymethyl-d_2_-2′-deoxycytidine-6-d_1_ (900 fmol), ^13^C_10_^15^N_2_-5-formyl-2′-deoxycytidine (500 fmol), and ^13^C_10_^15^N_2_-5-carboxyl-2′-deoxycytidine (300 fmol) (internal standards for mass spectrometry) and enzymatically digested to nucleosides as previously reported by Seiler et al.^12^ fC was derivatized with O-(biotinylcarbazoylmethyl) hydroxylamine (Cayman Chemical), and the digests were separated by offline HPLC using an Atlantis T3 column (Waters). Fractions containing 5mC, 5hmC, fC, and caC were collected, concentrated under vacuum, and analyzed using a Dionex Ultimate 3000UHPLC (Thermo Fisher, Waltham MA) interfaced with a Thermo TSQ Vantage mass spectrometer (Thermo Fisher) using a Zorbax SB-C18 column (0.5 × 150 mm, 3 µm, Agilent). Accurate quantitation was achieved in selected reaction monitoring mode using isotope dilution HPLC-ESI-MS/MS. Methods were fully validated as described in Supplementary Methods and shown in Fig. S2.

### Histone acetylation analyses

Acetylation stoichiometry of H3K14, H3K23, and H4K16 was determined using a mass spectrometry based quantitative methodology developed in our group^35^.

### Methylation specific PCR (MSP) and quantitative reverse transcription–PCR (qRT-PCR)

These experiments were conducted by standard methods as described in Supplementary Methods and elsewhere^7,12^.

### Methylation analysis by pyrosequencing

DNA isolated from mouse lung tissues (100 ng) was treated with bisulfite using an EpiTect Bisulfite Kit (Qiagen, Frederick MD) according to the manufacturer’s instructions. DNA was amplified by PCR with primers for the following genes: *Ahrr, DAPK1, CDH13, Tet1, and Rassf1*. Bisulfite converted DNA was prepared for pyrosequencing according to the instructions in the PyroMark assay kit (Qiagen, Frederick, MD). Pyrosequencing was carried out according to the design files from Qiagen and the Qiagen PyroMark Assay Design SW 2.0 on the PyroMark Q96 (Qiagen, https://www.qiagen.com/us/products/discovery-and-translational-research/epigenetics/dna-methylation/pyrosequencing/software/pyromark-supplementary-software/). Primer sequences and experimental details are given in the Supplementary Methods.

### RRBS and oxRRBS

DNA was prepared for RRBS and oxRRBS using the Ovation RRBS Methyl-Seq system with TrueMethyl oxBS module (NuGEN, Redwood City, CA) according to the manufacturer’s protocol. Library amplification was optimized as directed using qRT-PCR and the libraries were amplified accordingly followed by Agencourt bead clean-up. Libraries were quantified using the PicoGreen dsDNA assay (Thermo Fisher), and library size distribution was evaluated using the Bioanalyzer High Sensitivity assay (Agilent). Paired-end sequencing (2 × 75 bp) was performed on an Illumina NextSeq 550 instrument (Illumina, San Diego, CA) using a 150-cycle High-Output flow cell kit at the University of Minnesota Genomics Center. A custom Read 1 sequencing primer was used (MetSeq Primer 1) along with the standard Illumina Read 1 primer. RRBS and oxo-RRBS reads were trimmed with TrimGalore! version 0.4.4_dev, and the Cleaned reads were aligned to the mm10 reference genome with Bismark version 0.19.0^55^. Analysis of methylated and hydroxymethylated regions followed the Methpipe analysis pipeline^17^. Differentially methylated (DMR) and hydroxymethylated (DhMR) regions were identified by merging consecutive CpGs that crossed the threshold for statistical significance at a false discovery rate of 0.05, and filtering regions with fewer than three significant CpG sites. Gene bodies that overlapped with DMRs and DhMRs were used in Ingenuity Pathway Analysis (IPA). Details of RRBS/oxo-RRBS methods and data analysis are given in Supplementary Methods.

*Detailed Methods, MSP and PCR primer sequences, representative HPLC–ESI–MS/MS traces, HPLC–ESI–MS/MS validation curves, pyrosequencing results, and qRT-PCR data are presented in the Supplementary Information.*

## Supplementary information

Supplementary file1

Supplementary file2
