# Supplementary material for: Inhalation exposure to cigarette smoke and inflammatory agents induces epigenetic changes in the lung
Source: Sci Rep. 2020 Jul 9;10:11290. doi: 10.1038/s41598-020-67502-8 (PMC7347915; doi:10.1038/s41598-020-67502-8)
Supplement: Supplementary file 1 — Supplementary file1 [file 41598_2020_67502_MOESM1_ESM.docx]

April 15, 2020

**Supplemental Information for:**

**Inhalation Exposure to Cigarette Smoke and Inflammatory Agents Induces Epigenetic Changes in the Lung**

Christopher L. Seiler,^†,£^ Jung Min Song,^‡,£^ Delshanee Kotandeniya,^†,£^ Jianji Chen,^¥^ Thomas J. Y. Kono,^κ^ Qiyuan Han, ^¥^ Mathia Colwell,^γ^ Benjamin Auch,^β^ Aaron L. Sarver,^¶,£^ Pramod Upadhyaya,^£^ Yanan Ren,^α^ Christopher Faulk,^γ^ Silvio De Flora,^€^ Sebastiano La Maestra,^€^ Yue Chen,^¥^ Fekadu Kassie,^‡,£^ and Natalia Y. Tretyakova^†,£,*^

^†^Department of Medicinal Chemistry, ^‡^Department of Veterinary Medicine, ^¥^Department of Biochemistry, ^κ^Minnesota Super Computing Institute, ^γ^Department of Animal Science, ^β^University of Minnesota Genomics Center, ^¶^Institute for Health Informatics, ^α^Biostatistics Core, and ^£^Masonic Cancer Center, University of Minnesota, Minneapolis, Minnesota 55455. ^€^Department of Health Sciences, University of Genoa, 16132 Genoa, Italy.

**Table of Contents:**

Methods 4

Table S1. MSP primer sequences employed in this study. 16

Table S2. Primer sequences used for qRT-PCR. 17

Figure S1. Representative capillary HPLC-ESI-MS/MS traces for accurate quantification of MeC, hmC, fC, and caC in mouse DNA. 18

Figure S2. HPLC-ESI-MS/MS validation curves for MeC, hmC, and fC. Fixed amounts of isotopically labeled internal standards (1 pmol 13C1015N2-MeC, 900 fmol d3-hmC, and 500 fmol 13C1015N2-fC) were spiked into calf thymus DNA and along with increasing amounts of the corresponding unlabeled nucleosides. Samples were processed as described above and subjected to HPLC-ESI+-MS/MS analysis. 19

Figure S3. Epigenetic DNA marks and enzymatic pathways involved in their formation. 20

Figure S4. Global amounts of fC in lung DNA of mice exposed to cigarette smoke for 10 weeks with or without aspirin co-treatment. 21

Figure S5. NNK and LPS alter the global levels of epigenetic modifications, MeC and hmC, in the lung tissue of treated A/J mice. 22

Figure S6. Early changes in global MeC and hmC levels in kidney and brain DNA of A/J mice treated with NNK and/or LPS. 23

Figure S7. Global changes in genomic MeC, hmC, fC, and caC levels in the brain of A/J mice chronically treated with NNK, LPS, or both NNK and LPS for 5 weeks. 24

Figure S8. Methylation status of specific CpG sites within promoter regions of Ahrr, DAPK1, CDH13, Tet1, and Rassf1 genes in lung DNA of mice treated for 6 weeks with vehicle, NNK, LPS, and NNK/LPS determined by bisulfite pyrosequencing. 25

Figure S9. Gene expression changes in the lung tissues of A/J mice treated with a single dose of NNK (100 mg/kg, IP) in week 1, weekly LPS (5 µg/mouse in 50 µL PBS, intranasally under isoflurane anesthesia) starting week 2, both NNK and LPS or physiological saline only (control) for a total of 9 weeks. 26

Figure S10. Methylation specific PCR results from DNA isolated from lung tumors of A/J mice treated for 44 weeks with a single dose of NNK (100 mg/kg, IP) in week 1 and biweekly dosing of LPS (2 µg/mouse in 50 µL PBS, intranasally under isoflurane anesthesia) beginning in week 2. 27

Figure S11. Gene expression changes in the lung tumors of A/J mice treated with a single dose of NNK (100 mg/kg, IP) in week 1, biweekly LPS (2 µg/dose in 50 µL PBS, intranasally under isoflurane anesthesia) starting week 2, or both NNK/LPS for a duration of 44 weeks as compared to normal lung tissue control. 28

Figure S12. Scatterplot showing the methylation and hydroxymethylation fractions of CpG sites within DMRs or DhMRs. 29

Figure S13. Barplot showing the distribution of distance from DMR/DhMR to transcription start site...30

Methods

**Chemicals and Enzymes.** PDE I, PDE II, and DNase I were purchased from Worthington Biochemical Corp. (Lakewood, NJ), while calf intestinal alkaline phosphatase was from Sigma-Aldrich, (Madison, WI). Nanosep10K filters were acquired from Pall corp. (Port Washington, NY). O-(biotinylcarbazoylmethyl) hydroxylamine was obtained from Cayman Chemical (Ann Arbor, MI). Isotopically labeled D_3_-hmC was purchased from Cambridge Isotope Labs (Cambridge, MA). ^13^C_10_,^15^N_2_-5-methyl-2ʹ-deoxycytidine was synthesized as previously described.^1^ All other chemicals were bought from Sigma-Aldrich (Milwaukee, WI) or Fisher Scientific (Fairlawn, NJ).

**Animal Treatments.**

***Cigarette smoke exposure treatment (Fig. 1a, part 1):***

This study was conducted at the University of Genoa, Italy. Newborn, female A/J mice were divided into two groups (4 per group). Group 1 was exposed to filtered air for the duration of the study, and group 2 was exposed to environmental cigarette smoke starting 12 hours after birth. Mice were euthanized in a CO_2_ chamber. The lungs were isolated, and DNA was extracted from the left lung and stored at -20 ℃ until analysis.

***Acute Treatment of mice with NNK, LPS and NNK/LPS (Fig. 1a, part 2)***

Female A/J mice were obtained from the Jackson Laboratory (Bar Harbor, ME) and housed in specific-pathogen-free animal quarters at Research Animal Resources, University of Minnesota Academic Health Center. All animal experiments were performed according to the U.S. National Institutes of Health (NIH) Guide for the Care and Use of Laboratory Animals and was approved by the Institutional Animal Care and Use Committee, University of Minnesota.

Female A/J mice (6 weeks of age) were divided into 3 groups (3 animals per group). Mice in Group 1 were treated intraperitoneally (IP) with NNK (25 mg/kg, in 0.3 ml physiological saline solution) every day for 3 – 9 days and 12.5 mg/kg NNK daily on days 10-15 days. For lipopolysaccharide (LPS) treatments, 8.3 µg of LPS was administered intranasally twice a week in the first week of the experiment, and 4.15 µg LPS was given once a week during the second week. Mice were euthanized in a CO_2_ chamber the day after NNK treatment or allowed to recover for 1 week. Tissues (lung, brain, kidney) were harvested and stored frozen at -80 °C until analysis.

***Subchronic Treatment with NNK, LPS, and NNK/ LPS (Fig. 1a, part 3).*** Female A/J mice (6 weeks of age) were divided into four groups: control (N = 4), NNK (N = 5), LPS (N = 4), and NNK+LPS (N = 4). NNK was administered IP (50 mg/kg) twice a week during weeks 1 and 3, and once during week 5 for a total of 5 doses. LPS was given intranasally (4 µg/mouse) twice in week 1, and once a week during weeks 3, 4, and 5, for a total of 5 doses. Control mice were treated with physiological saline IP for the same duration of time. Mice were euthanized in a CO_2_ chamber during the sixth week of treatment. Tissues (lung and brain) were harvested and stored at -80 °C until analysis.

***Lung Tumor induction with NNK/LPS:*** Female A/J mice (7 weeks of age) were divided into three groups (20 per group). Group 1 was treated intraperitoneally (IP) with NNK (100 mg/kg, in 0.3 ml PBS) once a week for two weeks, group 2 was treated intranasally with 4 µg of LPS (in 50 µL of 1 X PBS) a week after the 2^nd^ dose of NNK administration and subsequently treated with the same dose of LPS once a week until week 27. Mice in control group 3 were given physiological saline (0.3 mL, IP). Mice were euthanized in a CO_2_ chamber. Tumors (≥ 100) were pooled from lung lobes of 4-5 mice in group 1 and 2 and stored frozen at -80 °C until analysis.

**Synthesis of Stable Isotope Labeled Internal Standards**

**^13^C_10_^15^N_2_-5-formyl-2′-deoxycytidine**: To the stirring solution of ^13^C_10_^15^N_2_-5-methyl-2′-deoxycytidine (1 mg, synthesized previously)^1^ in a 1 M phosphate buffer at pH 7 was added sodium persulfate (16.4 mg). The mixture was placed in a preheated oil bath and stirred at 70 °C for 3 days. The reaction mixture was purified by RP-HPLC using a Synergi Hydro-RP (250 mm x 4.6 mm x 4 µm, Phenomenex, Torrance CA) with a linear gradient of acetonitrile and water (3% to 5% over 25 min), with product eluting at 16.8 minutes.

**^13^C_10_^15^N_2_-5-carboxyl-2′-deoxycytidine**: ^13^C_10_^15^N_2_-5-formyl-2′-deoxycytidine (57 µg, 210 nmol) was placed in an Eppendorf tube along with potassium monophosphate (16 mg, Sigma-Aldrich), isoamylene (82 µL, Sigma-Aldrich), sodium chlorite (16 mg, Sigma-Aldrich), water (180 µL), tetrahydrofuran (444 µL, Fisher Scientific), and t-butanol (800 µL, Fisher Scientific). The reaction was stirred for 2.5 hours at room temperature and then acidified with 400 µL of 1 N HCl. The reaction mixture was separated by RP-HPLC using a gradient of 0.1% formic acid in water and 0.1% formic acid in acetonitrile on a Synergi Hydro-RP column (250 mm x 4.6 mm x 4 µm, Phenomenex) with a linear increase from 3% to 5% acetonitrile and ^13^C_10_^15^N_2_-cadC eluted at 7 min.

**DNA Digestion and Enrichment of MeC, hmC, fC, and caC**

Genomic DNA was extracted from lung, kidney, brain tissues and lung tumors using IBI-Mini Genomic DNA Kit (IBI Scientific, Peosta IA) according to the manufacturer’s protocol. DNA concentrations were determined by UV spectroscopy.

Genomic DNA (2-10 µg) was subjected to hydrolysis with PDE I (3.6 U, 5 µg), PDE II (3.2 U), DNase I (50U), and alkaline phosphatase (10 U) in 10 mM Tris HCl/15 mM MgCl_2_ buffer (pH 7) at 37 °C overnight. The hydrolysates were spiked with ^13^C_10_^15^N_2_-5-methyl-2′-deoxycytidine (1 pmol), 5-hydroxymethyl-d_2_-2′-deoxycytidine-6-d_1_ (900 fmol), ^13^C_10_^15^N_2_-5-formyl-2′-deoxycytidine (500 fmol), and ^13^C_10_^15^N_2_-5-carboxyl-2′-deoxycytidine (300 fmol) (internal standards for mass spectrometry synthesized in our laboratory)^1^ and filtered through Nanosep 10K Omega filters (Pall Corporation, Port Washington, NY).

DNA hydrolysates were dissolved in 100 mM ammonium acetate buffer (pH 4.5) containing 100 mM aniline and 400 µM O-(biotinylcarbazoylmethyl) hydroxylamine (Cayman Chemical, Ann Arbor, MI) and allowed to react for 24 h to derivatize fC to biotinyl-fC.^2^ The resulting mixture was dried, followed by offline HPLC to enrich for MeC, hmC, biotinyl-fC, and caC using an Atlantis T3 column (Waters, 4.6 x 150 mm, 3 µm) was eluted at a flow rate of 0.9 mL/min with a gradient of 5 mM ammonium formate buffer, pH 4.0 (A) and methanol (B). Solvent composition was changed linearly from 3 to 30% B over 15 min, increased to 80% over the next 3 min, maintained at 80% B for the next 2 min, and brought back to 3% B. The column was equilibrated for 7 min. dC was quantified by HPLC-UV using calibration curves obtained by analyzing authentic dC standards. HPLC fractions corresponding to MeC, hmC, caC, and biotinyl-fC (7-8.6 min for both hmC and caC, 9-10.5 min for MeC, and 18.7-20.2 min for biotinyl-fC) were combined, dried, and analyzed by isotope dilution HPLC-ESI-MS/MS.

**HPLC-ESI^+^-MS/MS quantitation of MeC, hmC, and fC**

Quantitation of MeC, hmC, and biotinyl-fC was performed using a Dionex Ultimate 3000UHPLC (Thermo Fisher, Waltham MA) interfaced with a Thermo TSQ Vantage mass spectrometer (Thermo Fisher) as previously reported by Seiler *et. al*.^3^ Chromatographic separation was achieved on a Zorbax SB-C18 column (0.5 x 150 mm, 3 µm, Agilent) eluted at a flow rate of 15 μL/min with a gradient of 2 mM ammonium formate (A) and methanol (B). Solvent composition was maintained at 5% B for the first 3 min and linearly changed from 5 to 40% B for 7 min. Solvent composition was a returned to initial conditions (5% B), and the column was re-equilibrated for 4 min. Under these conditions, MeC and ^13^C_10_^15^N_2_-MeC eluted at 4.1 min, both hmC and the internal standard d_3_-hmC eluted at 3.5 min, while biotinyl-fC and its internal standard (^13^C_10_^15^N_2_-biotinyl-fC) eluted at 7.0 min. Quantitation was achieved by monitoring the transitions *m/z* 258.1 [M + H^+^] → *m/z* 141.1 [M – deoxyribose + H^+^] for hmC, *m/z* 261.2 [M + H^+^] → *m/z* 145.1 [M – deoxyribose + H^+^] for D_3_-hmC, *m/z* 242.1 [M + H^+^] → *m/z* 126.1 [M + H^+^] for MeC, *m/z* 254.2 [M + H^+^] → *m/z* 133.1 [M + H^+^] for ^13^C_10_^15^N_2_-MeC, *m/z* 569.1 [M + H^+^] → *m/z* 453.3 [M – deoxyribose + H^+^] for Biotinyl-5fC, *m/z* 581.2 [M + H^+^] → *m/z* 460.4 [M – deoxyribose + H^+^] for Biotinyl-^13^C_10_^15^N_2_-5fC. Mass spectrometer were determined by infusion of authentic standards. Typical MS settings were: a spray voltage of 2700 V, a sheath gas of 15 units, the declustering voltage was 5 V, and the ion transfer tube was maintained at 350 ℃. The full-width at half-maximum (FWHM) was maintained at 0.7 for both Q1 and Q3. Fragmentation was induced using a collision gas of 1.0 mTorr and a collision energy of 10.3 V.

**HPLC-ESI^-^-MS/MS analyses of caC**

caC was quantified in the negative ion mode by monitoring the transitions *m/z* 270.00 [M - H^+^] → 109.97 [M – deoxyribose – CO_2_ - H^+^] for caC and *m/z* 282.00 [M - H^+^] → 116.05 [M – deoxyribose – CO_2_ - H^+^] for ^13^C_10_^15^N_2_-caC internal standard. Chromatographic separation was achieved on a Thermo Hypercarb column (0.5 x 100 mm, 3 µm, Thermo Fisher Scientific) eluted at a flow rate of 14 μL/min with a gradient of 2.5 mM ammonium bicarbonate (A) and acetonitrile (B). Solvent composition was increased from 10 to 15% B over the first 3 min and further to 60% B over 7 min. Solvent composition was returned to initial conditions over 1 min and re-equilibrated over 6 min. Under these conditions, caC and ^13^C_10_^15^N_2_-caC eluted at 5.5 minutes. MS parameters were optimized to achieve maximum sensitivity. The mass spectrometer was operated in negative mode with an S-lens voltage of 72 V, a spray voltage of -3.0 kV, capillary temperature at 270 °C, declustering voltage of 7 V, and nitrogen as a sheath gas at 20 arbitrary units. CID was achieved at with collision energy of 18 V. Argon was used as a collision gas with a pressure of 1.0 mTorr. MS/MS analyses were performed with a scan width of 0.1 *m/z* and a scan time of 0.1.

**HPLC-ESI^+^-MS/MS method validation**

Fixed amounts of isotopically labeled internal standards (1 pmol ^13^C_10_^15^N_2_-MeC, 900 fmol d_3_-hmC, and 500 fmol ^13^C_10_^15^N_2_-fC) and increasing amounts of the corresponding unlabeled nucleosides (2 pmol – 200 pmol MeC, 100 fmol – 20 pmol hmC, and 1 fmol – 500 fmol fC) were spiked into 1 µg of commercial calf thymus DNA (Sigma). Samples were processed by enzymatic hydrolysis, ultrafiltration, and offline HPLC as described above and subjected to HPLC-ESI-MS/MS analysis. The observed amounts of MeC, hmC, and fC were plotted against theoretical values followed by linear regression analysis (**Fig. S2**).

The LOD and LOQ values of the new method were determined by spiking synthetic dsDNA (5 µg, sequence: 5′-AGCTTATCGCAGCCGGCGCGAATCTGA-3′) with increasing amounts of synthetic oligodeoxynucleotides each containing a single MeC, hmC, or fC residue (5′-AGCTTATCGCAGC**X**GGCGCGAATCTGA-3′, where X = MeC, hmC, or fC) and fixed amounts of internal standards (1 pmol ^13^C_10_^15^N_2_-MeC, 900 fmol d_3_-hmC, and 300 fmol ^13^C_10_^15^N_2_-fC), followed by sample processing and capillary HPLC-ESI-MS/MS analysis as described as above. The LOD values were determined as the lowest analyte amounts that consistently produced signal-to-noise ratios above 3. The LOQ was defined as the minimum amount of analyte that produced a coefficient of variation less than 15% and a signal-to-noise ratio greater than 10.

To evaluate precision and inter-day and intra-day accuracy of the method, samples were processed as above and analyzed three times per day on three consecutive days. Accuracy was calculated for each analyte using the equation: A_m_/A_a_ x 100%, where A­_m_ is the measured amount of analyte and A_a_ is the amount of analyte added.

**Histone acetylation analysis**

Lung tissues from control and treated mice were homogenized with a glass douncer on ice. Total histones were extracted from the lysates as previously described.^4^ Histone proteins were acetylated with (^13^C_2_,d_3_)-acetyl N-hydroxysuccinimide ester to block all unmodified lysines and then digested by trypsin.^5^ The peptides were desalted with C18 Stage Tips (3M Corporation, St. Paul, MN) prior to analysis.

Tryptic peptides were analyzed by nano-flow liquid chromatography electrospray tandem mass spectrometry (nanoLC-ESI-MS/MS) using a Thermo Scientific Orbitrap Fusion mass spectrometer (Thermo Scientific, San Jose, CA) coupled to a Proxeon Easy nLC 1000 UPLC system (Thermo Fisher Scientific, Odense, Denmark). Each sample was re-suspended in HPLC buffer A (0.1% formic acid in water) and loaded onto an in-house packed C18 column (25 cm x 75 μm I.D.) packed with ReproSil-Pur Basic C18 beads (2.5 μm, Dr. Maisch GmbH). Peptides were eluted with a gradient of 5% to 15% B (0.1% formic acid in acetonitrile) over 26 minutes, then 15% to 35% HPLC buffer B over 16 minutes at 300 nL/min.

Tryptic peptides from histone proteins were analyzed using a FT survey scan from 300-1600 *m/z* at a resolution of FWHM 120,000 (at 200 *m/z*), followed by HCD MS/MS scans using the top speed mode (3 seconds per cycle) at a resolution of FWHM 15,000 (at 200 m/z) and the normalized collision energy at 35%. The targeted MS/MS data acquisition was achieved with an inclusion list for fully labeled histone tryptic peptides that covered known lysine acetylation sites. For each peptide, modification isomers with all possible combinations of light/heavy lysine acetylation (delta mass of 42.010565 and 47.036094 Da, respectively) at detectable charge states were considered in the inclusion list for targeted fragmentations.

MS data was searched against the Uniprot Mus musculus proteome database (http://www.uniprot.org) using MaxQuant search engine (v1.4.1.2) as previously described.^282-284^ Heavy and light acetylation on lysine as well as methionine oxidation were included as variable modifications, with 6 ppm specified as the precursor mass error and 0.025 Da as the fragment mass error. All peptide spectra matches were filtered at 1% False Discovery Rate with a minimum Andromeda score cutoff of 40. The HPLC elution profile were manually evaluated to ensure accurate quantifications. Only peptides that were confidently identified were selected for stoichiometry analysis. Acetylation stoichiometries of specific sites were calculated using in-house developed scripts based on the extracted peak areas of each modification isomer and quantification of modification-specific fragment ions.^1^ Statistical significance analysis of site-specific acetylation stoichiometry dynamics between control and treated samples was conducted using two-sided Student’s t-test using SAS statistical software 9.3 (SAS Institute Inc., Cary, NC).

**Methylation specific PCR (MSP) assay**

Genomic DNA was isolated from mouse lung tissues or lung tumors using a QIAamp DNA mini kit (Qiagen, Valencia, CA). For bisulfite conversion, 0.5 μg of the isolated genomic DNA was treated with sodium bisulfite using an EpiTect Bisulfite kit (Qiagen, Valencia, CA).

Methylation specific PCR of bisulfite treated DNA was performed using EpiTect MSP kit (Qiagen, Valencia, CA) using methylated and unmethylated primer sets listed in **Table S1**. PCR reaction conditions were performed as follows: 1 cycle at 95°C for 10 min; 40 cycles at 94°C for 15 s, 48°C for 30 s, and 72°C for 30 s; 1 cycle at 72°C for 10 min. The resulting PCR products were analyzed on 2% agarose gels after staining ethidium bromide. Three human NSCLC cell lines (A549, H1299 and H2009) and a mouse cancer cell line (MCS) derived from mouse lung tumors induced were used as positive control samples to indicate the correct fragment by MSP.

**Quantitative reverse transcription–PCR (qRT-PCR) analysis**

Total RNA was extracted from frozen mouse lung or lung tumor tissues using the miRNeasy Mini Kit (Qiagen, Valencia, CA) according to the manufacturer’s instruction. The purity and the integrity of total RNA were confirmed by Nanodrop UV-Spectrophotometer and the RNA was stored at -80 ℃ until later use. The first-strand complementary DNA was synthesized by using QuantiTect Reverse Transcription Kit (Qiagen, Valencia, CA) with one microgram of RNA in 20 µL reaction. The first-strand complementary DNA mixture was further diluted to 200 µL with RNase-free water and stored at -20 °C until use.

qRT–PCR was performed by Light Cycler 96 (Roche, Indianapolis, IN) using QuantiTect SYBR Green PCR Kit (Qiagen, Valencia, CA) and gene specific primers (**Table S2**). Twenty-five nanograms of complementary DNA was added to a 20 µL reaction. The final concentration of each primer was 0.5 µM. For PCR amplification, a program of initial denaturation at 95 °C for 15 min, followed by 45 cycles consisting of denaturation at 94 °C for 15 s, annealing at 50 °C for 30 s and extension at 72 °C for 34 s was used. All samples were normalized to an internal control (β-actin). Comparative Ct method was used to assess the relative levels of gene expression. Values were expressed as relative units compared with vehicle control (mouse lung tissue) and the standard error.

**Methylation Analysis by Pyrosequencing**

DNA isolated from mouse lung tissues (100 ng) was treated with bisulfite using EpiTect Bisulfite Kit (Qiagen, Frederick MD) according to the manufacturer’s instructions. Bisulfite treated DNA was amplified by PCR with primers for the following genes: Ahrr, DAPK1, CDH13, Tet1, and Rassf1. The thermocycler protocol for amplification reactions consisted of 95°C for 30 sec, the optimal annealing temperature for 30 sec, and 72 °C for 30 sec, each reaction completing 40 rounds of PCR. PCR product amplicon sizes were checked on a QIAxcel (Qiagen) prior to pyrosequencing to ensure the correct gene was amplified. Bisulfite converted DNA was prepared for pyrosequencing according to the instructions in the PyroMark assay kit (Qiagen, Frederick, MD), including three controls: “no template control” (NTC), bisulfite converted 100% and 0% methylated mouse DNA. Methylated mouse DNA controls were created by methylation of genomic mouse DNA with M.SssI. Pyrosequencing was carried out according the design files from Qiagen and the Qiagen Assay Design Software on the PyroMark Q96 (Qiagen). CpG methylation values were used for statistical analysis, and values defined as “failed” were discarded. Statistical significance was calculated in GraphPad Prism 6 using 2-way ANOVA.

**RRBS and oxo-RRBS**

DNA isolated from lung tissues of mice exposed to environmental cigarette smoke was prepared for RRBS and oxo-RRBS using the Ovation RRBS Methyl-Seq System with TrueMethyl oxBS module (NuGEN, Redwood City, CA) according to the manufacturer’s protocol. Briefly, 100 ng of isolated DNA was fragmented using MspI at 37 °C for 1 h. Adapters were ligated to the fragmented DNA at 25 °C for 30 min, which was followed by repair at 60 °C for 10 min. DNA was cleaned up from the reaction mixture using Agencourt magnetic beads using a 2:1 bead:DNA solution ratio. Oxidation reactions were incubated at 40 °C for 10 min. Bisulfite conversion of DNA was performed using the reagents supplied by NuGEN and cleaned up using Agencourt beads. Library amplification was optimized as directed using qRT-PCR and the libraries were amplified accordingly followed by Agencourt bead clean-up.

Libraries were quantified using the PicoGreen dsDNA assay (ThermoFisher), and library size distribution was evaluated using the Bioanalyzer High Sensitivity assay (Agilent). The final library pool was also quantified and normalized using qPCR (KAPA Library Quantification Kit, Roche). Paired-end sequencing (2 x 75 bp) was performed on an Illumina NextSeq 550 instrument (Illumina, San Diego, CA) using a 150-cycle High-Output flow cell kit at the University of Minnesota Genomics Center. A custom Read 1 sequencing primer was used (MetSeq Primer 1) along with the standard Illumina Read 1 primer. An extended 12-cycle indexing read was performed to allow for duplicate read determination via random bases incorporated into the adapter.

**RRBS and oxo-RRBS data analysis**

RRBS and oxo-RRBS reads were trimmed of low-quality bases and adapter contamination with TrimGalore! version 0.4.4_dev in paired-end mode. Cutadapt version 1.8.1 was used for the adapter trimming routine in TrimGalore!. The adapter sequences used for trimming were AGATCGGGAAGAGC (Illumina universal adapter sequence) and AAATCAAAAAAAC (NuGEN Ovation RRBS Methyl-Seq adapter sequence). The trimming was performed with default error rate, stringency, minimum quality, and minimum length parameters. Read pairs in which one mate failed quality control were discarded; only complete pairs were used in the analysis. Cleaned reads were aligned to the mm10 reference genome with Bismark version 0.19.0.^6^ The alignment parameters were adjusted for higher sensitivity during alignment. The seed length was decreased to 15bp, allowing one mismatch. The minimum score allowed for a read to align was set to -0.6 * read length, with mismatches between the read the reference counting as the maximum mismatch penalty, regardless of the reported base quality. Resulting alignments were cleaned of PCR duplicates with NuDup (<https://github.com/nugentechnologies/nudup)>, and reads with mapping quality of less than 20 were removed.

Analysis of methylated and hydroxymethylated regions followed the Methpipe analysis pipeline.^7^ Bismark alignments were converted to the “methylated read” format for Methpipe input and sorted by coordinate. Bisulfite conversion rates were estimated with the ‘bsrate’ program in Methpipe. Methylated read files were used to generate methylation counts for all cytosines in the genome. Genome-wide cytosines were then filtered to represent only symmetric (CpG dinucleotide) sites. CpG sites from RRBS and oxo-RRBS data were used to estimate hydroxymethylation rates with the ‘mlml’ program in Methpipe.^8^ Methylation counts and hydroxymethylation counts for CpG sites were combined and filtered for coverage. CpG sites that did not have at least 10 reads in each sample were discarded. CpG sites were further filtered to exclude sites that occurred within “blacklisted” regions of the mm10 genome assembly (ENCODE file ID ENCFF547MET). The filtered methylation and hydroxymethylation tables were used to fit beta-binomial regression models to identify differentially methylated and hydroxymethylated regions with the ‘radmeth’ program in Methpipe.^9^ Local smoothing was applied in windows of 200bp with the ‘adjust’ routine of ‘radmeth.’ Differentially methylated (DMR) and hydroxymethylated (DhMR) regions were identified by merging consecutive CpGs that crossed the threshold for statistical significance at a false discovery rate of 0.05, and filtering regions with fewer than three significant CpG sites.

We then used bedtools version 2.27.0 to identify DMRs and DhMRs that overlapped with annotated genes, putative promoter elements, and enhancer elements.^10^ The annotated gene list was derived from the mm10 annotation hosted on Ensembl. The putative promoter element list was downloaded from the Eukaryotic Promoter Database, and promoter regions were treated as spanning the interval from 2000bp upstream of the transcription start site to 10bp downstream of the transcription start site, on the same strand as the annotated gene. Putative enhancer elements were downloaded from the VISTA enhancer database.

Gene bodies that overlap with DMRs were used in Ingenuity Pathway Analysis (IPA) core analysis, treating the mean of the individual-CpG Q-values as a FDR-adjusted P-value and mean methylation fraction difference as a normalized expression value. Promoters that overlap with DhMRs were also used for IPA core analysis. For all IPA analyses, the considered species was restricted to mouse, the cell types were restricted to lung tissues and immune cells.

Table S1. MSP primer sequences employed in this study.

| **MSP primer** | **Forward primer (5ʹ-3ʹ)** | **Reverse primer (5ʹ-3ʹ)** |
| --- | --- | --- |
| **mDAPK1** |  |  |
| Methylated | AGGAGTCGCGAGCGTAGC | CAACTATCGCGTACGC |
| Unmethylated | TGGGAGGAGTTGTGAGTGT | ACAACTATCACTTCATAC ACC |
| **mRAR-BETA** |  |  |
| Methylated | TCGTGGTTTTTTTGTGCGGTTC | CAACATACAAAAAAAAAAACTGCGG |
| Unmethylated | TTGTGGATTTTTTTGTGTGGTTTG | CAACATACAAAAAAAAAAACTCACAA |
| **mGATA2** |  |  |
| Methylated | ATTAGGTAGATAGGGCGTAGAGTTC | CTAACTATCTCTCGATTCCCGAC |
| Unmethylated | GATTAGGTAGATAGGGTGTAGAGTTTG | TTCTAACTATCTCTCAATTCCCAAC |
| **mCDH13** |  |  |
| Methylated | TATTTGTTATGTAAAACGAGGGAGC | CAAATAAATCAACGACAACATCG |
| Unmethylated | TTTGTTATGTAAAATGAGGGAGTGT | CCAAATAAATCAACAACATCAC |
| **mRUNX3** |  |  |
| Methylated | TGTAGTTATAAGATTTTTTAAGGGGTC | CACAAAATACAAAAAACCAACTCG |
| Unmethylated | GTAGTTATAAGATTTTTTAAGGGGTTGT | TCACAAAATACAAAAAACCAACTCA |
| **hDAPK1** |  |  |
| Methylated | GGGATTTTAGTATATATTTCGGGAC | GAACTACCCTACCAAACCGA |
| Unmethylated | TTGGGATTTTAGTATATATTTTGGGAT | CAAACTACCCTACCAAACCA |
| **hRAR-BETA** |  |  |
| Methylated | GGTTAGTAGTTCGGGTAGGTTTTATC | CCGAATCCTACCCCGACG |
| Unmethylated | TTAGTAGTTTGGGTAGGGTTTATT | CCAAATCCTACCCCAACA |
| **hGATA2** |  |  |
| Methylated | CGGGTATTTTTTTGTTTTTTGC | TAACCTCGCTACCTTCCTAACG |
| Unmethylated | TTTTGGGTATTTTTTTGTTTTTTGT | CTAACCTCACTACCTTCCTAACACT |
| **hCDH13** |  |  |
| Methylated | AAGAAGTAAATGGGATGTTATTTTC | AAAACCAATAACTTTACAAAACGAA |
| Unmethylated | TTAAAGTAAATGGGATGTTATTTTT | ACCAAAACCAATAACTTTACAAAACA |
| **hRUNX3** |  |  |
| Methylated | GGTTTAGTTAATGAGTTAAGGTCGC | TCTAATAAATACGAAAACGACCGA |
| Unmethylated | TTTAGTTAATGAGTTAAGGTTGTGA | TCTAATAAATACAAAAACAACCAA A |

Table S2. Primer sequences used for qRT-PCR.

| **Gene name** | **Forward primer (5ʹ-3ʹ)** | **Reverse primer (5ʹ-3ʹ)** |
| --- | --- | --- |
| mDAPK1 | CCGCTGTCAACTACGACTTT | GTCCTGGATTGTCATCCTCTTC |
| mRAR-β | CCTCTGACTGACCTTGTGTTC | GGCGGTCTCCACAGATTAAA |
| mGATA2 | GACGACAACCACCACCTTAT | TGCTGGACATCTTCCGATTC |
| mCDH13 | CCTGACAAGCCATCTCCTAAC | GACATCCAATCCTGCCATATCT |
| mPRDM2 | TAGGTCCCGTGTGTGTATCT | CTGCTTTCCCATCACTCTGT |
| mRUNX3 | GAGTTTCACGCTCACAATCAC | GCCTTGGTCTGGTCTTCTATC |
| mRASSF1 | GAGACACCCGATCTTTCTCAAG | CACTGAAACAGGACGCACTA |
| mβ-actin | ACTCTTCCAGCCTTCCTTCC | GTACTTGCGCTCAGGAGGAG |
| mTet1 | AGATGGCTCCAGTTGCTTATC | CTTCCGTTGTGCATGTTGTG |
| mTet2 | GTCCTGATGTGGCAGCTATT | TCCTCACTCGATCTCCGATATAC |
| mTet3 | GAGTTCCCTACCTGCGATTG | TCCATGAGTTCCCGGATAGA |

Figure S1. Representative capillary HPLC-ESI-MS/MS traces for accurate quantification of MeC, hmC, fC, and caC in mouse DNA.


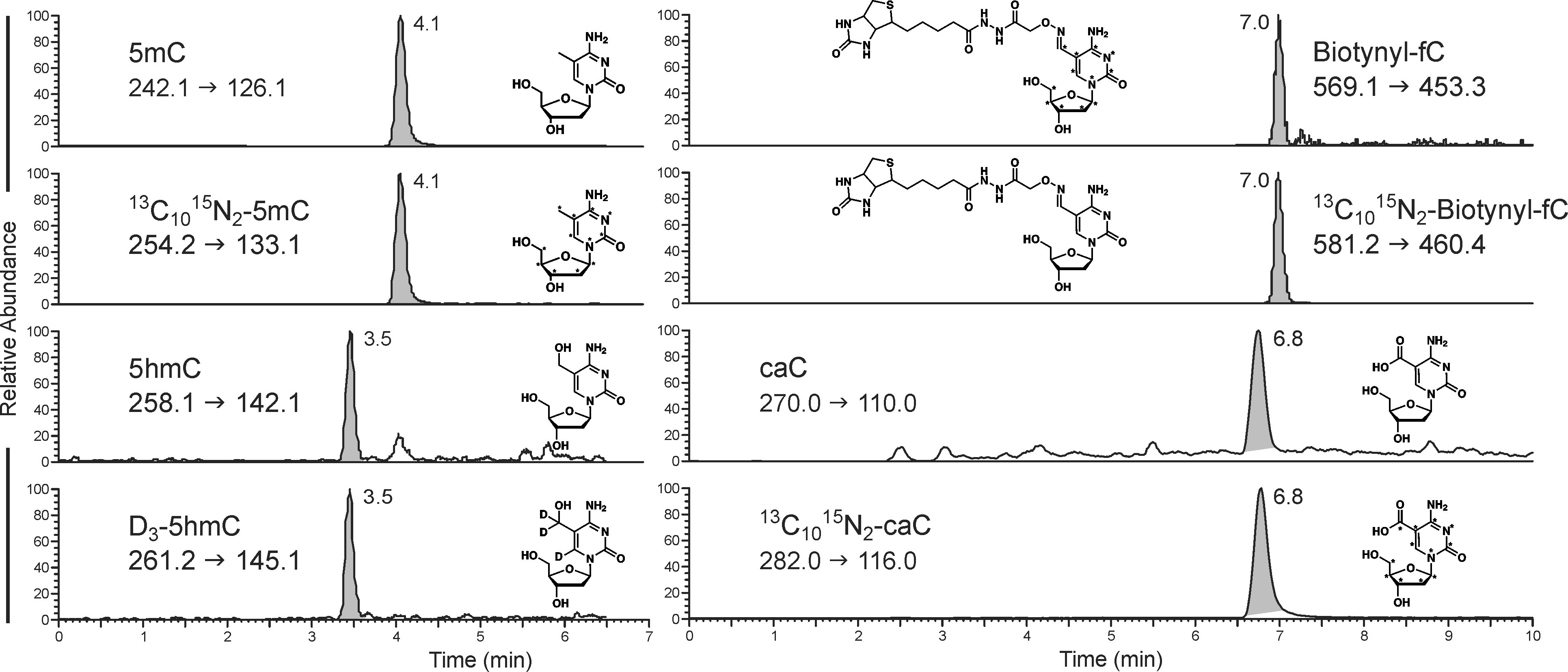


Figure S2. HPLC-ESI-MS/MS validation curves for MeC, hmC, and fC. Fixed amounts of isotopically labeled internal standards (1 pmol ^13^C_10_^15^N_2_-MeC, 900 fmol d_3_-hmC, and 500 fmol ^13^C_10_^15^N_2_-fC) were spiked into calf thymus DNA and along with increasing amounts of the corresponding unlabeled nucleosides. Samples were processed as described above and subjected to HPLC-ESI^+^-MS/MS analysis.


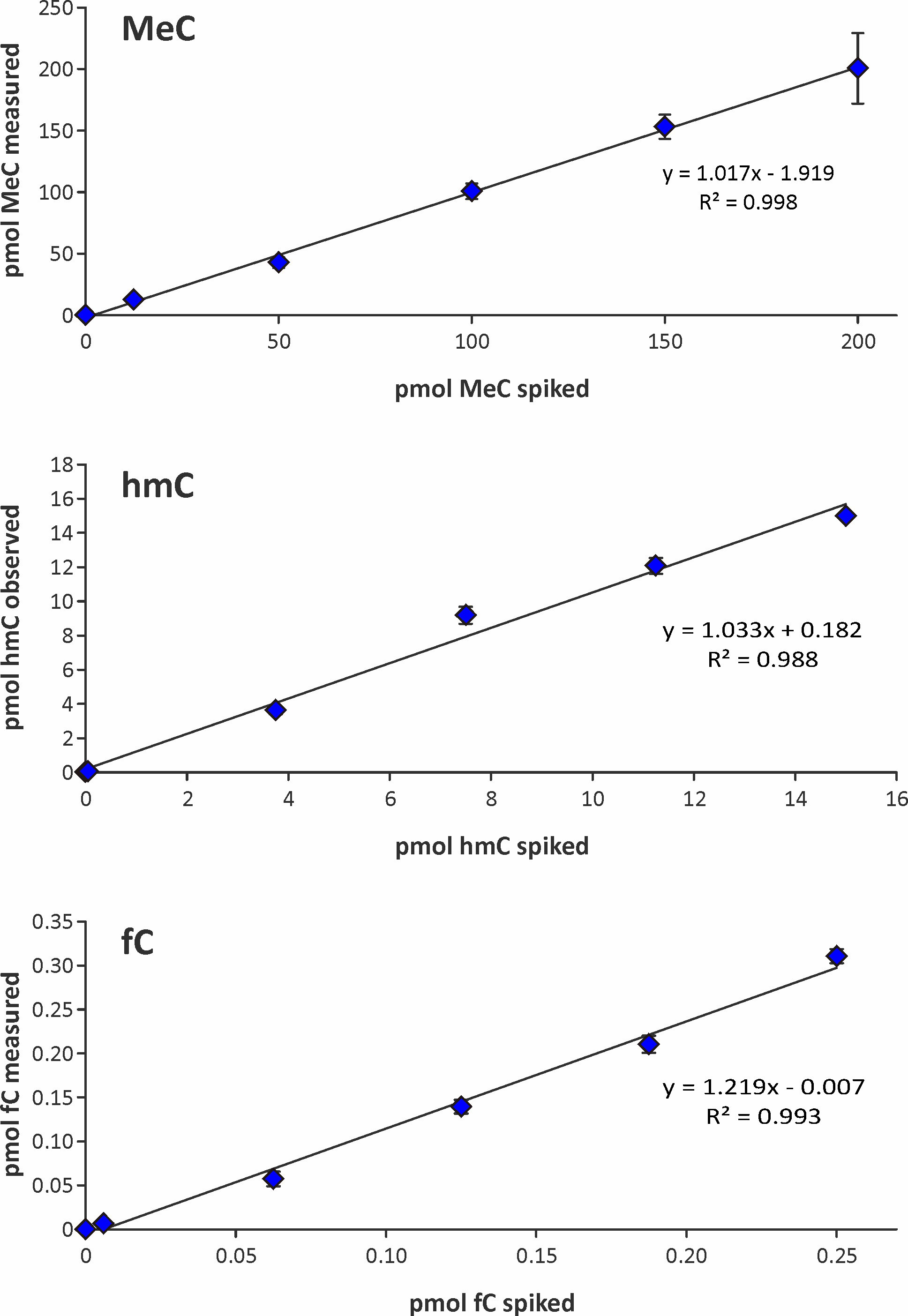


Figure S3. Epigenetic DNA marks and enzymatic pathways involved in their formation. Formation of MeC from cytosine is catalyzed by DNMT enzymes. Oxidation of MeC to hmC, fC, and caC is carried out by TET dioxygenases and can lead to DNA demethylation.


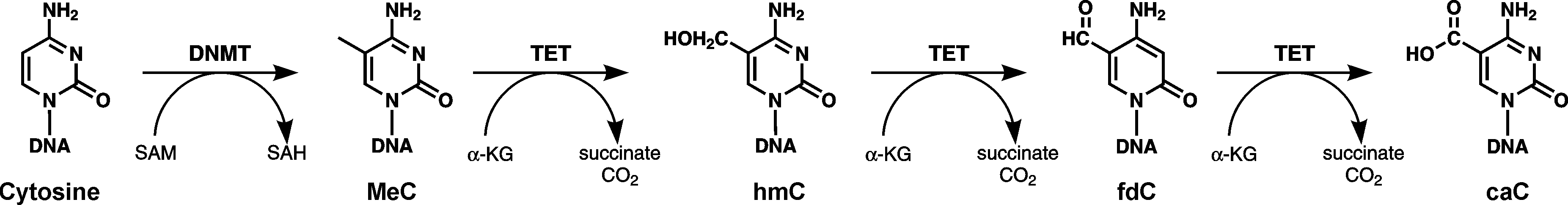


Figure S4. Global amounts of fC in lung DNA of mice exposed to cigarette smoke for 10 weeks with or without aspirin co-treatment.

**
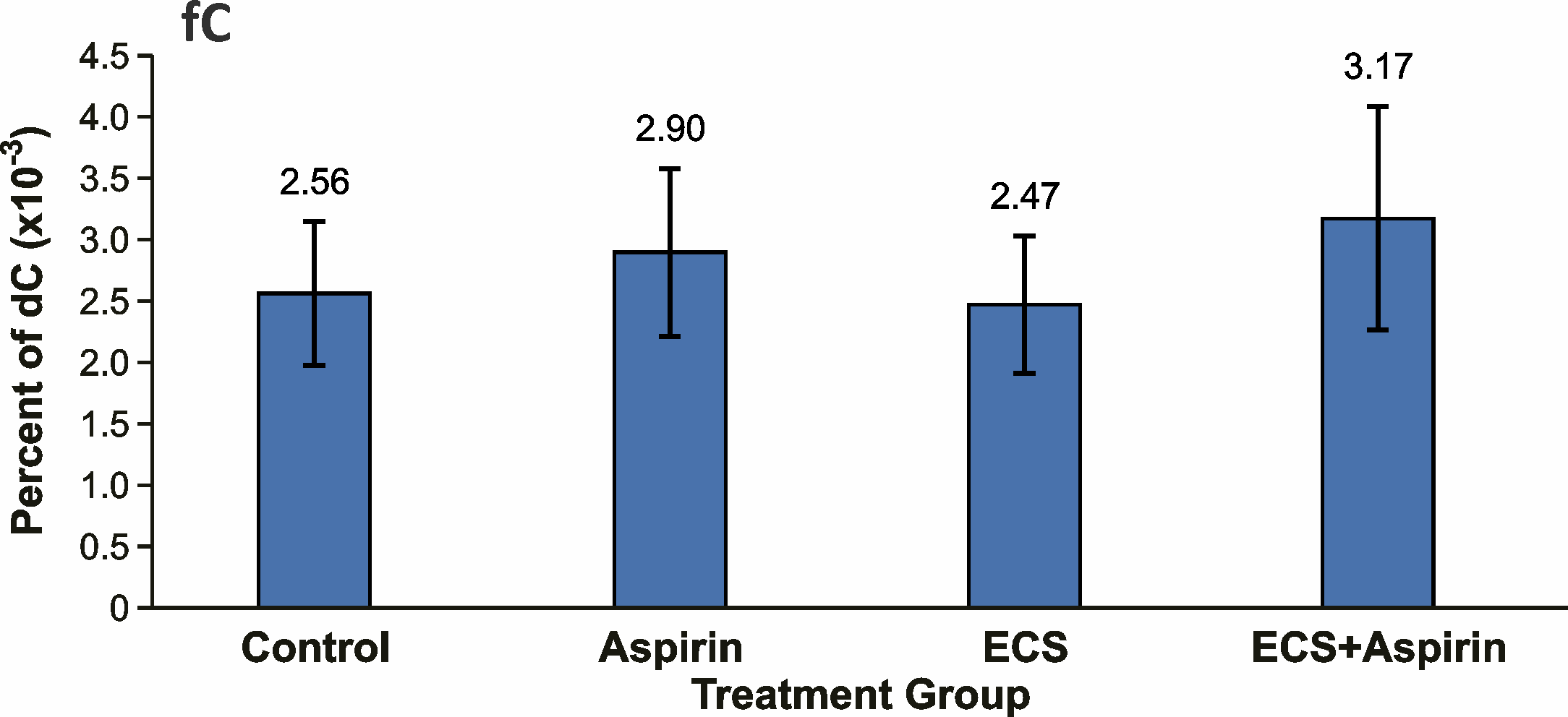
**

Figure S5. NNK and LPS alter the global levels of epigenetic modifications, MeC and hmC, in the lung tissue of treated A/J mice. Female A/J mice (6 weeks of age) were treated with NNK, with LPS, or both for a length of 2 weeks. Data are expressed as percent of dC and represents mean values ± SD of at least three animals. Global levels of MeC and hmC in the lung tissue of mice treated for 2 weeks with NNK or in combination with LPS showed little change for the levels of MeC, however a significant decrease was observed in hmC in the combination of NNK and LPS and in the mice sacrificed a week after the final treatment.


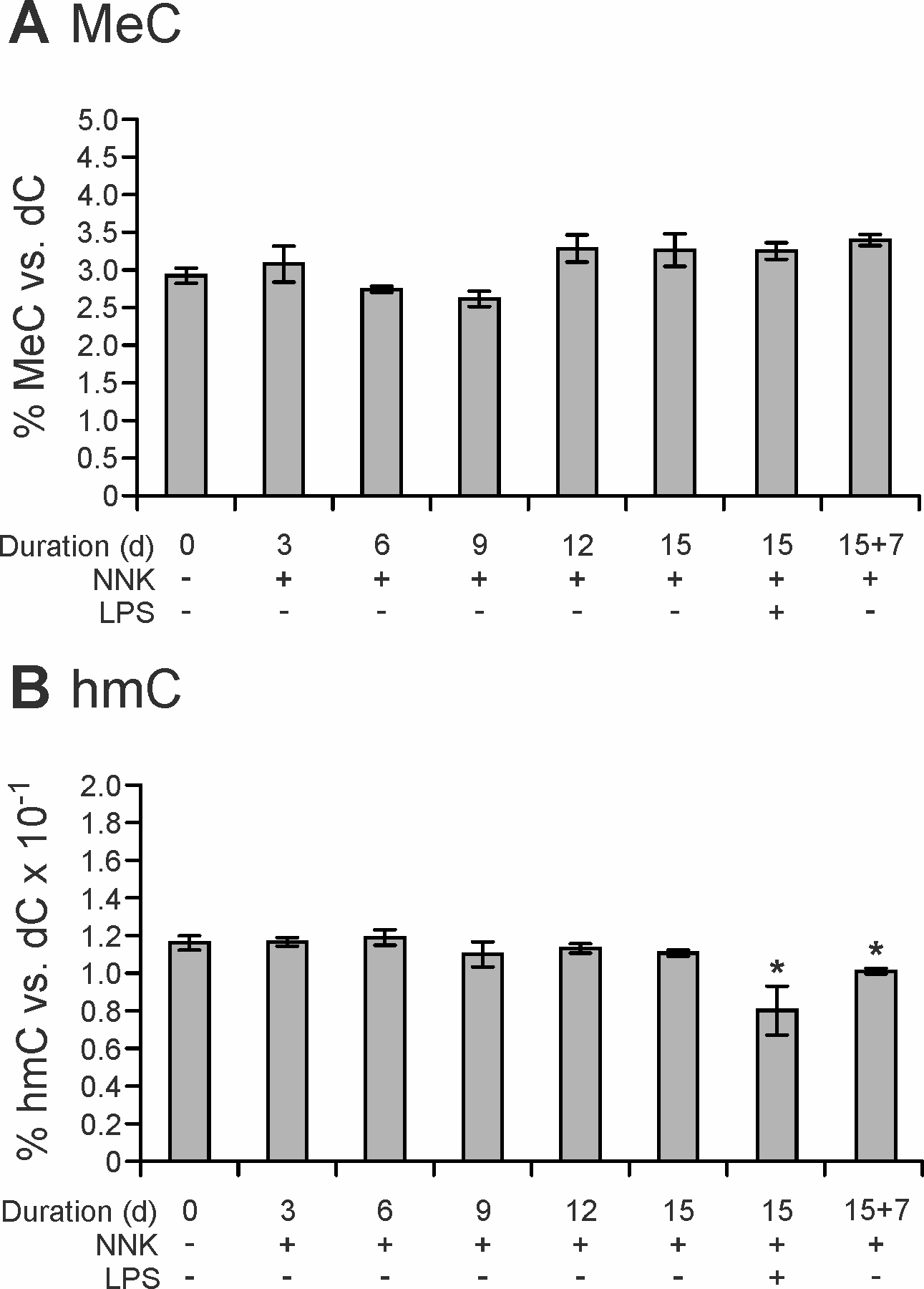


Figure S6. Early changes in global MeC and hmC levels in kidney and brain DNA of A/J mice treated with NNK and/or LPS. Female A/J mice (6 weeks of age) were treated intraperitoneally with NNK (25 mg/kg) every day on days 3 -9 and 12.5 mg/kg of NNK on days 10-14. For LPS treatments, 8.3 µg LPS was administered intranasally twice a week in the first week and 4.15 µg once during the second week. The error bars represent the average of N = 4 repeats for LPS-control and N = 3 for the rest of the groups.


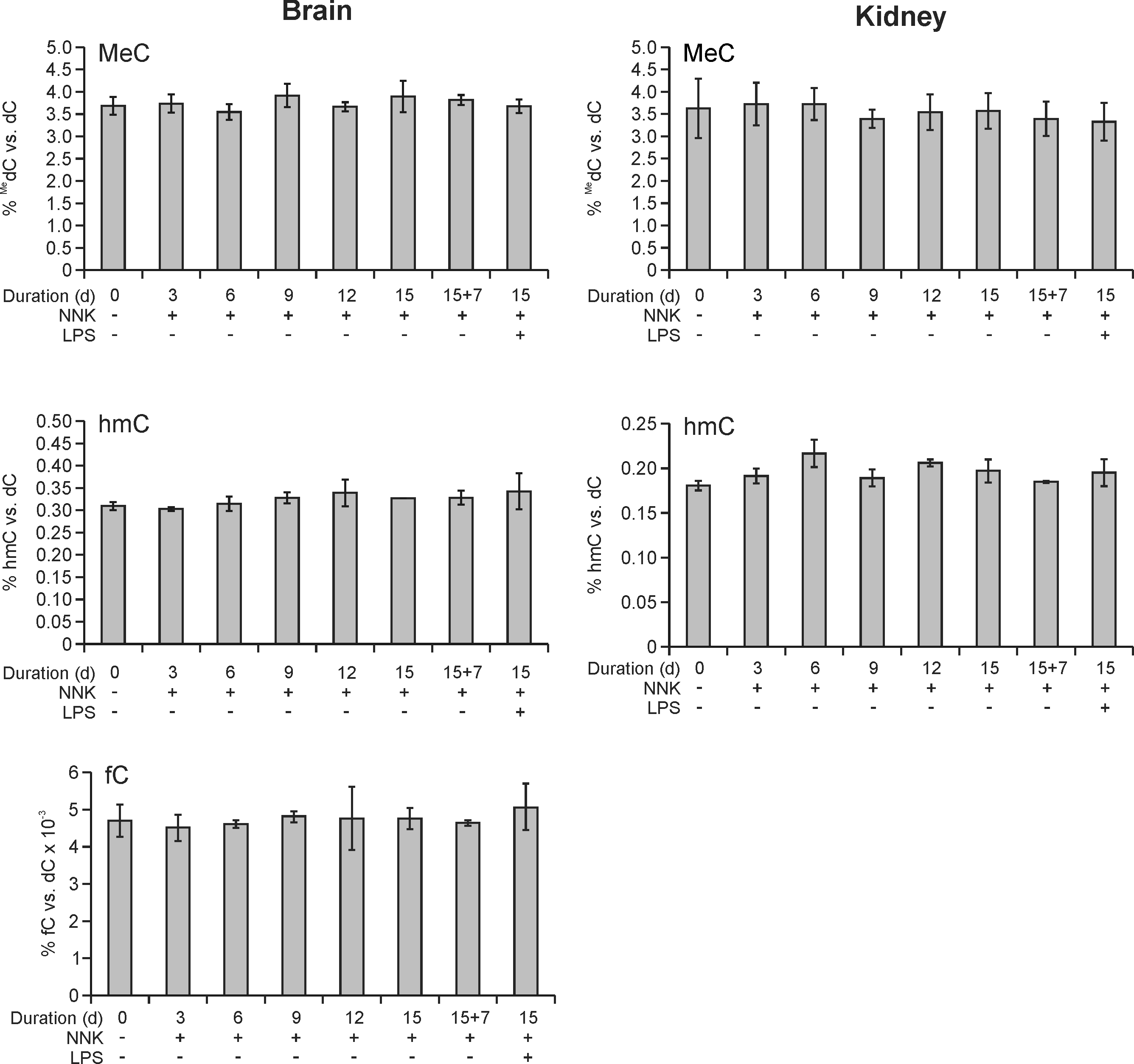


Figure S7. Global changes in genomic MeC, hmC, fC, and caC levels in the brain of A/J mice chronically treated with NNK, LPS, or both NNK and LPS for 5 weeks. NNK (50 mg/kg) was given IP twice during weeks 1 and 3, and once during week 5. LPS (4 µg/mouse) was administered intranasally twice during week 1, and once a week in weeks 3, 4, and 5. The error bars represent the average of N = 4 repeats.


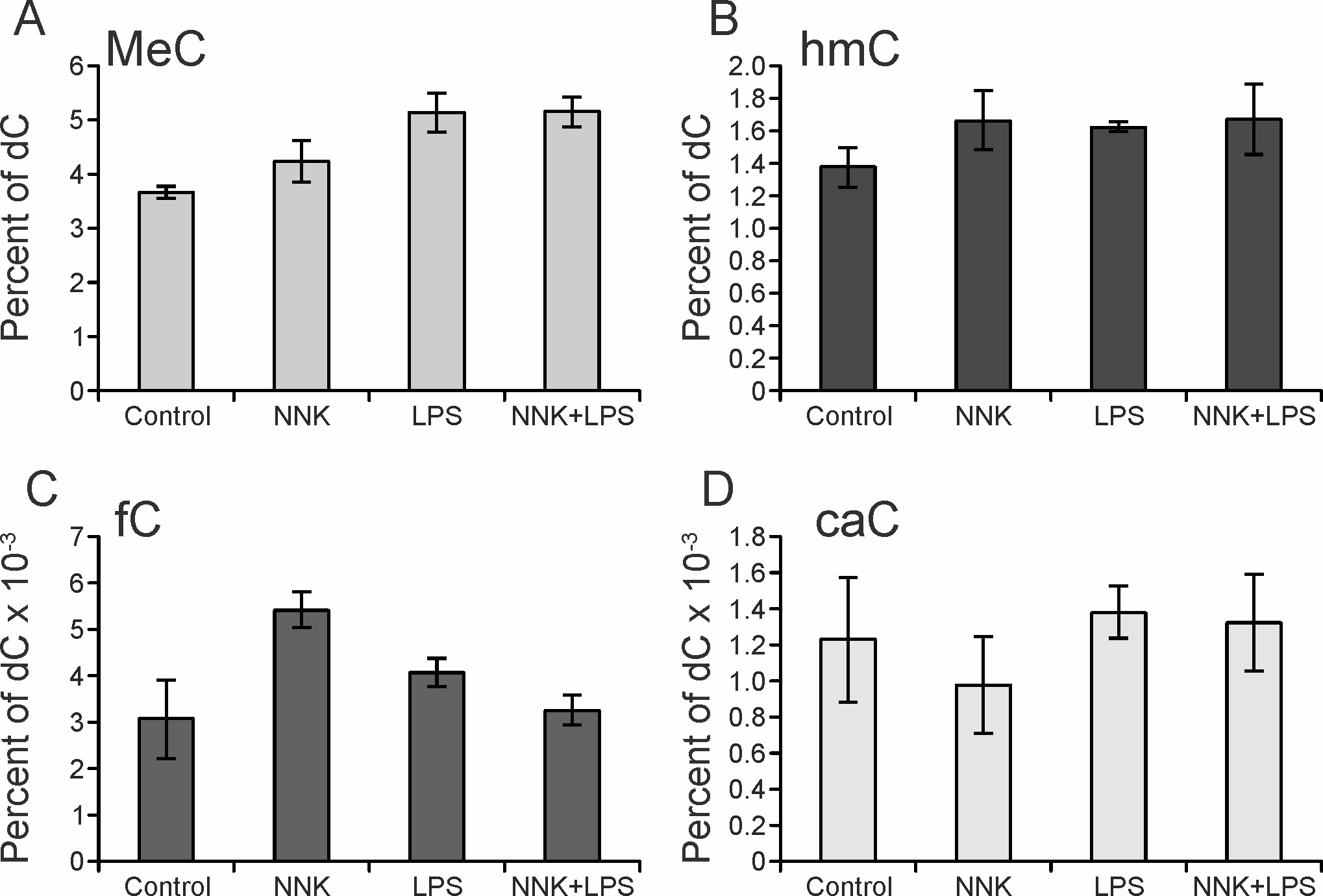


Figure S8. Methylation status of specific CpG sites within promoter regions of Ahrr, DAPK1, CDH13, Tet1, and Rassf1 genes in lung DNA of mice treated for 6 weeks with vehicle, NNK, LPS, and NNK/LPS determined by bisulfite pyrosequencing. * indicates p < 0.05 vs. control.


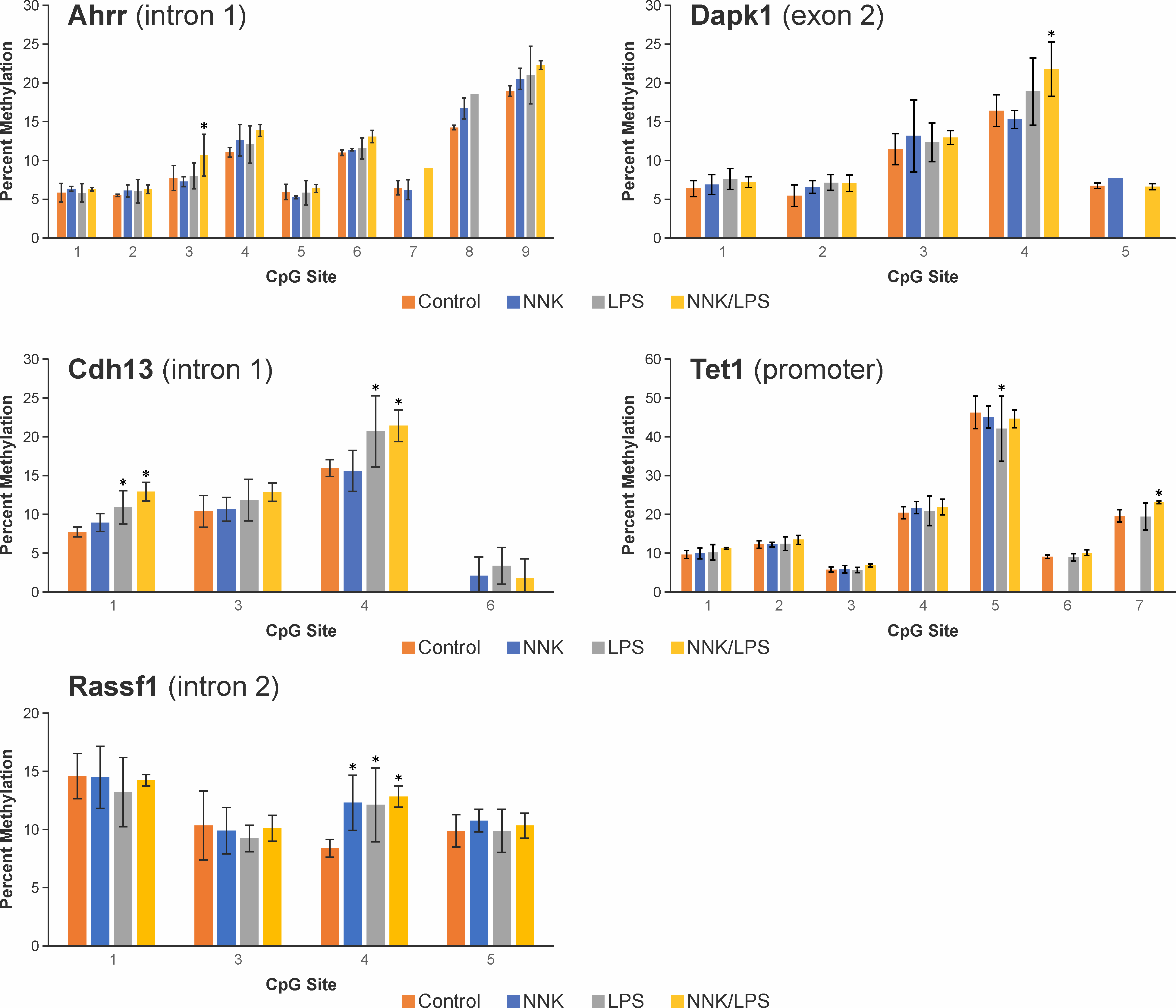


Figure S9. Gene expression changes in the lung tissues of A/J mice treated with treated with a single dose of NNK (100 mg/kg, IP) in week 1, weekly LPS (5 µg/mouse in 50 µL PBS, intranasally under isoflurane anesthesia) starting week 2, both NNK and LPS or physiological saline only (control) for a total of 9 weeks. qRT-PCR data were processed using the ΔΔCt method ± SD with three biological and three technical replicates. Genes were selected based on their association with lung cancer.


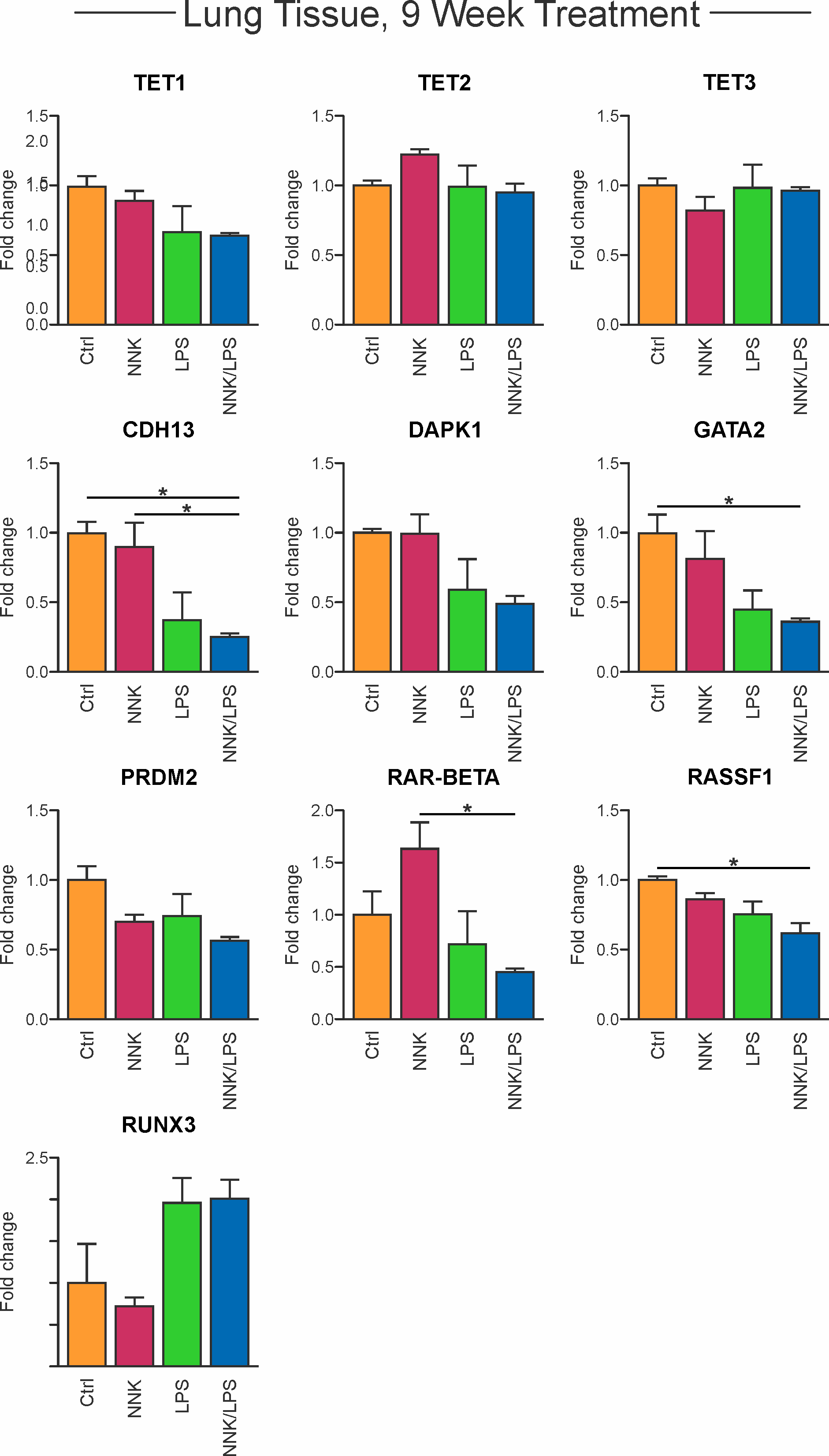


Figure S10. Methylation specific PCR results from DNA isolated from lung tumors of A/J mice treated for 44 weeks with a single dose of NNK (100 mg/kg, IP) in week 1 and biweekly dosing of LPS (2 µg/mouse in 50 µL PBS, intranasally under isoflurane anesthesia) beginning in week 2.

**
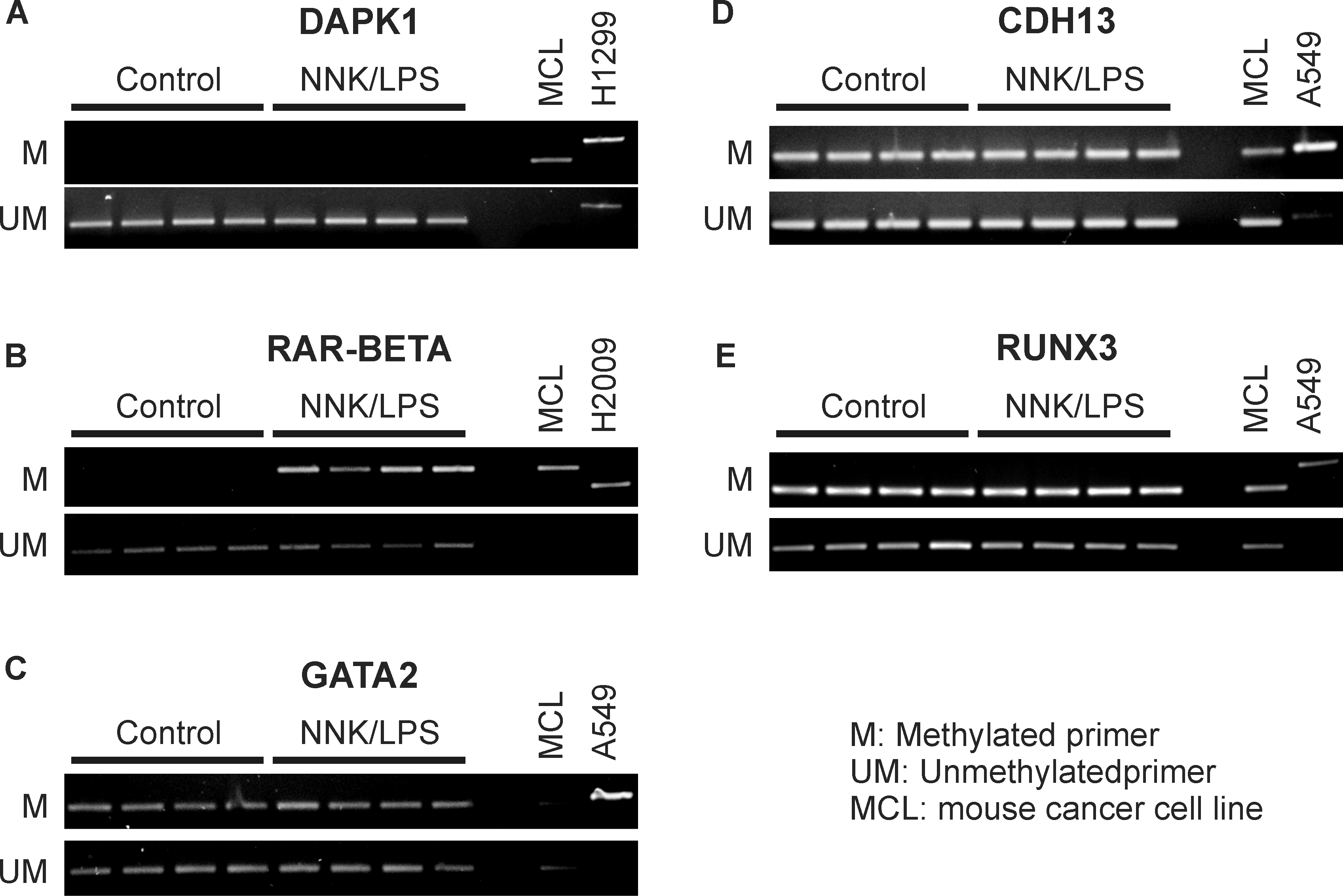
**

Figure S11. Gene expression changes in the lung tumors of A/J mice treated with a single dose of NNK (100 mg/kg, IP) in week 1, biweekly LPS (2 µg/dose in 50 µL PBS, intranasally under isoflurane anesthesia) starting week 2, or both NNK/LPS for a duration of 44 weeks as compared to normal lung tissue control. Data are calculated using the ΔΔCt method ± SD with three biological and three technical replicates.

**
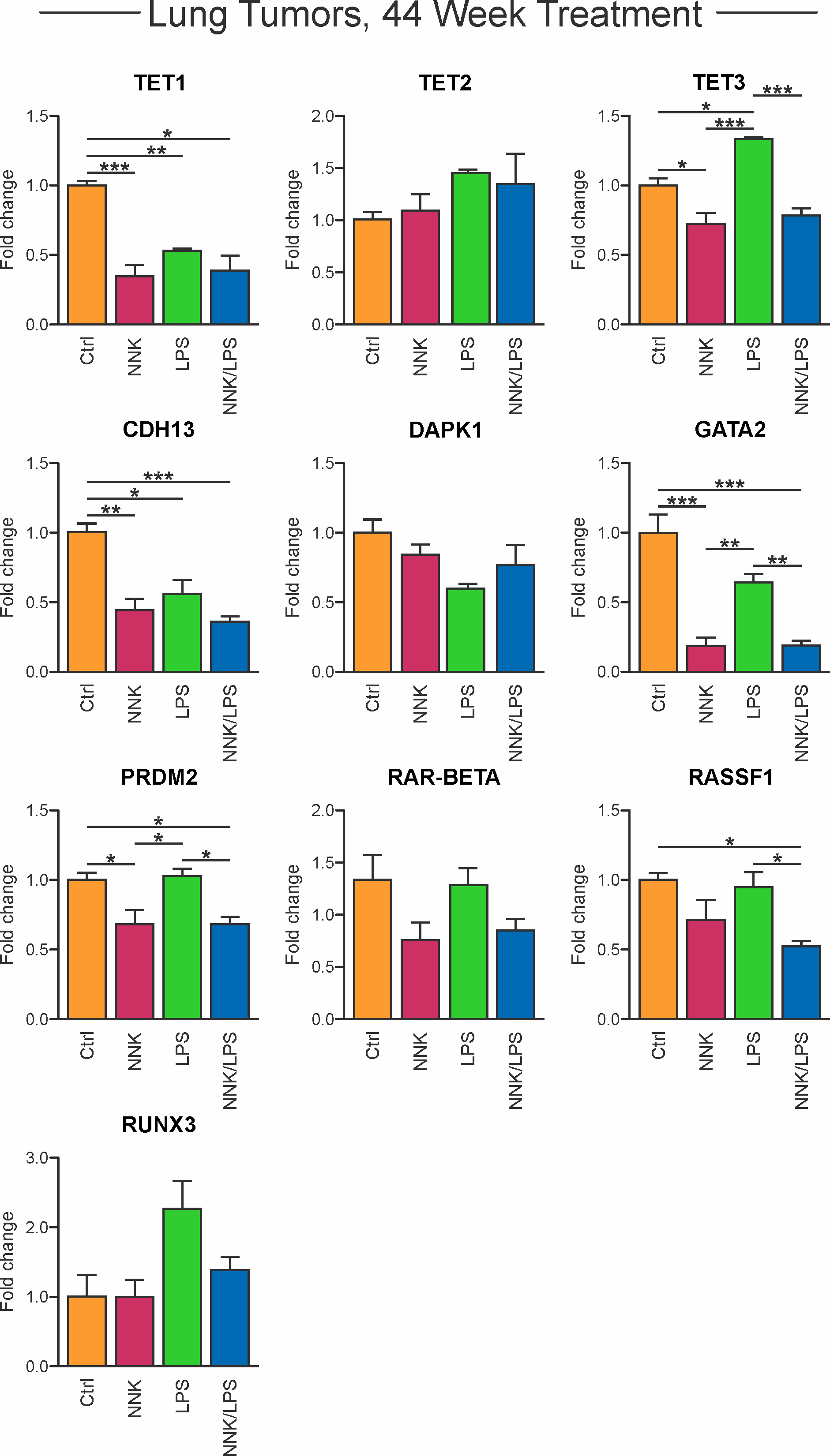
**

Figure S12. Scatterplot showing the methylation and hydroxymethylation fractions of CpG sites within DMRs or DhMRs. CpGs with sufficient coverage are represented by individual points. The solid lines are lowess curves fit to the data. CpGs in DMRs or DhMRs identified between treatment and control groups have slightly higher hydroxymethylation levels than methylation levels, on average.


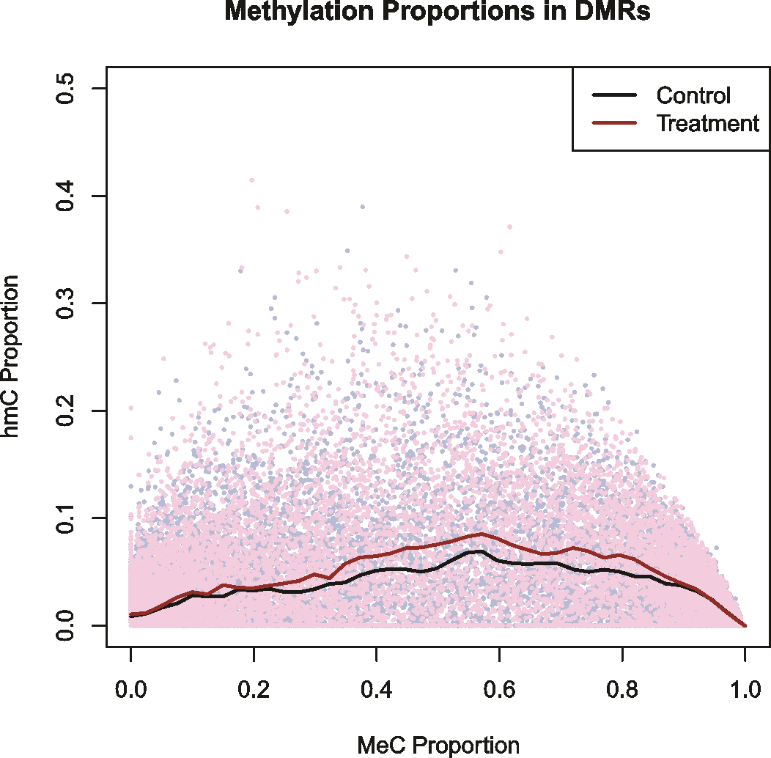


**Figure S13.** Barplot showing the distribution of distance from DMR/DhMR to transcription start site.

D(h)MRs at different distance ranges to TSS are represented by different colors. The percentage of D(h)MRs falling into different distance ranges are plotted across x axis around TSS.


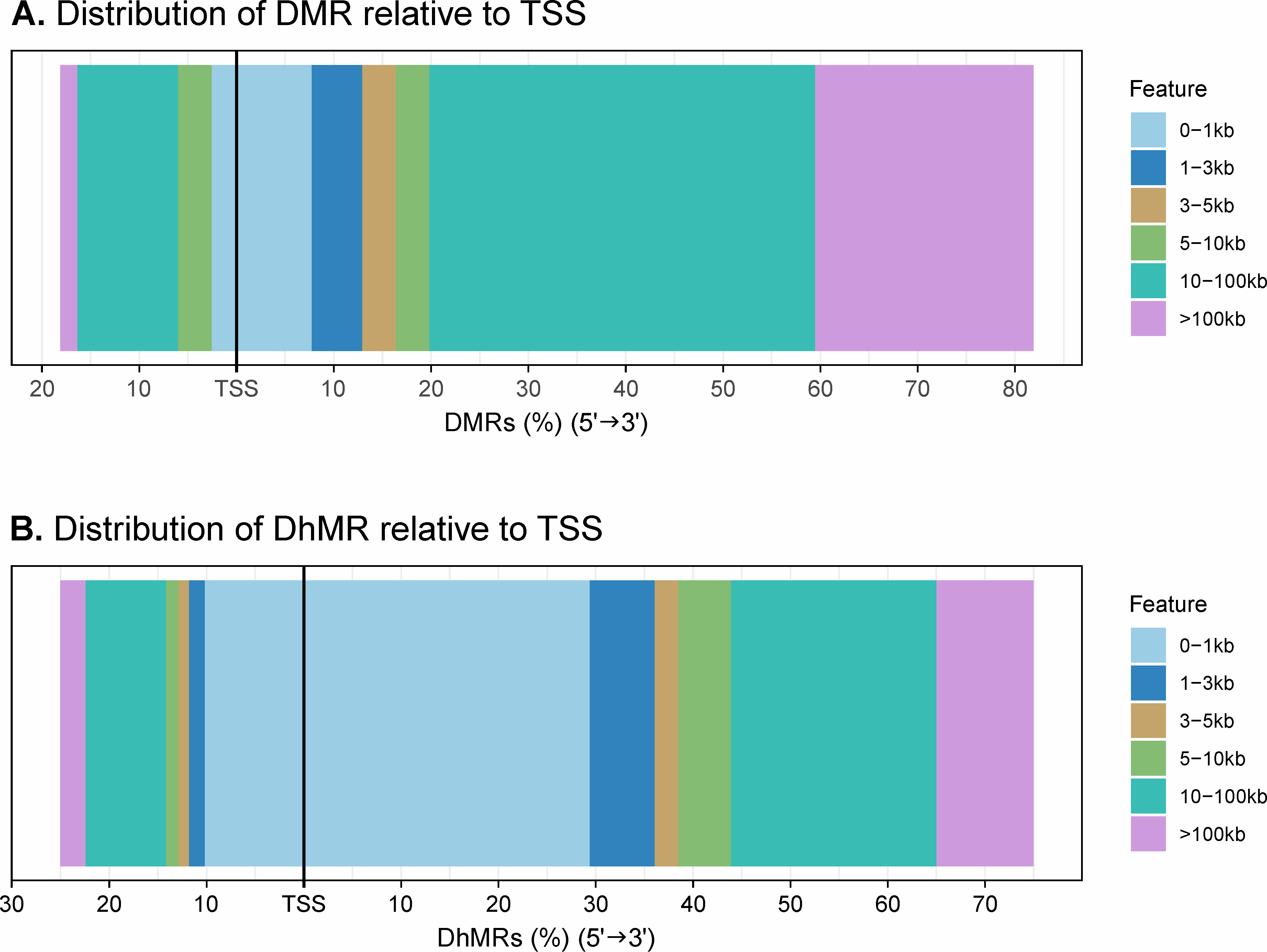


## **References**

1 Kotandeniya, D. *et al.* Can 5-methylcytosine analogues with extended alkyl side chains guide DNA methylation? *Chem Commun (Camb)* **54**, 1061-1064, doi:10.1039/c7cc06867k (2018).

2 Pfaffeneder, T. *et al.* The discovery of 5-formylcytosine in embryonic stem cell DNA. *Angew. Chem. Int. Ed Engl* **50**, 7008-7012 (2011).

3 Seiler, C. L. *et al.* Epigenetic changes in alveolar type II lung cells of A/J mice following intranasal treatment with lipopolysaccharide. *Chem Res Toxicol* **32**, 831-839, doi:10.1021/acs.chemrestox.9b00015 (2019).

4 Shechter, D., Dormann, H. L., Allis, C. D. & Hake, S. B. Extraction, purification and analysis of histones. *Nat Protoc* **2**, 1445-1457, doi:10.1038/nprot.2007.202 (2007).

5 Zhou, T., Chung, Y. H., Chen, J. & Chen, Y. Site-specific identification of lysine acetylation stoichiometries in mammalian cells. *J Proteome Res* **15**, 1103-1113, doi:10.1021/acs.jproteome.5b01097 (2016).

6 Krueger, F. & Andrews, S. R. Bismark: a flexible aligner and methylation caller for Bisulfite-Seq applications. *Bioinformatics* **27**, 1571-1572 (2011).

7 Song, Q. *et al.* A reference methylome database and analysis pipeline to facilitate integrative and comparative epigenomics. *PLoS One* **8**, e81148, doi:10.1371/journal.pone.0081148 (2013).

8 Qu, J., Zhou, M., Song, Q., Hong, E. E. & Smith, A. D. MLML: consistent simultaneous estimates of DNA methylation and hydroxymethylation. *Bioinformatics* **29**, 2645-2646, doi:10.1093/bioinformatics/btt459 (2013).

9 Dolzhenko, E. & Smith, A. D. Using beta-binomial regression for high-precision differential methylation analysis in multifactor whole-genome bisulfite sequencing experiments. *BMC Bioinformatics* **15**, 215, doi:10.1186/1471-2105-15-215 (2014).

10 Quinlan, A. R. & Hall, I. M. BEDTools: a flexible suite of utilities for comparing genomic features. *Bioinformatics* **26**, 841-842, doi:10.1093/bioinformatics/btq033 (2010).
